# Glioblastoma: Pathogenesis and Current Status of Chemotherapy and Other Novel Treatments

**DOI:** 10.3390/cancers12040937

**Published:** 2020-04-10

**Authors:** Vilashini Rajaratnam, Mohammad Mohiminul Islam, Maixee Yang, Rachel Slaby, Hilda Martinez Ramirez, Shama Parveen Mirza

**Affiliations:** Department of Chemistry & Biochemistry, University of Wisconsin-Milwaukee, 3210 N. Cramer Street, Milwaukee, WI 53201, USA

**Keywords:** glioblastoma, molecular targets, pathogenesis, chemotherapy, novel therapy

## Abstract

Glioblastoma is one of the most common and detrimental forms of solid brain tumor, with over 10,000 new cases reported every year in the United States. Despite aggressive multimodal treatment approaches, the overall survival period is reported to be less than 15 months after diagnosis. A widely used approach for the treatment of glioblastoma is surgical removal of the tumor, followed by radiotherapy and chemotherapy. While there are several drugs available that are approved by the Food and Drug Administration (FDA), significant efforts have been made in recent years to develop new chemotherapeutic agents for the treatment of glioblastoma. This review describes the molecular targets and pathogenesis as well as the current progress in chemotherapeutic development and other novel therapies in the clinical setting for the treatment of glioblastoma.

## 1. Introduction

Gliomas refer to all forms of intra-axial tumors that originate from glial cells of the central nervous system (CNS). They are the most common type of CNS tumors, representing about 80% of all malignant brain tumors [1,2]. Historically, they include types of cells that share similar histological characteristics, such as astrocytomas (high-grade astrocytomas are denominated glioblastomas), brain stem gliomas, ependymomas, oligodendrogliomas, optic pathway gliomas, and mixed gliomas [3,4]. This method of categorization helps to understand the histological features of gliomas; however, it does not provide information on the malignancy of a tumor. Meanwhile, rapid exploration in the past decade has provided significant insight not only for understanding the mechanisms of the neoplasm on a molecular basis, but also in designing new anticancer treatments. Therefore, in 2014, the International Society of Neuropathology included molecular information on top of the histological characteristics in brain tumor diagnoses [5,6,7]. This led to substantial modifications to the World Health Organization Classification of Tumors of the CNS (CNS WHO) in 2016 [5,6]. The updated CNS WHO further classified gliomas into grades (Grade I, II, III, and IV) based on pathological evaluation using molecular information on the malignancy level of the neoplasm. This subcategorization is particularly influential in clinical settings, as it can assist in determining the type of treatment(s) for patients. Grade I tumors are neoplasms with low proliferation rates that can be cured by surgery alone. On the other hand, grade II tumors are invasive and often recur despite low proliferative potential. Grade III tumors are generally malignant tumors with histological confirmation that exhibit anaplasia and rapid mitotic cell division, while grade IV gliomas are of the most advanced grade and are malignant tumors that have the poorest prognosis, with high potential for fatal outcome [5,6,8].

The most common and yet most deleterious grade IV glioma subtype is glioblastoma [9]. According to the Central Brain Tumor Registry of the United States (CBTRUS) Statistical Report 2011–2015, glioblastomas constitute about 57% of the average annual age-adjusted incidence rate of all neuroepithelial tumors and about 48% of all malignant brain and CNS tumors. It has been noted that the incidence rate of glioblastoma tumors is 1.58 times higher in the male population compared to females in the United States [1]. Despite aggressive multimodal treatment, due to the detrimental nature and quick progression (median survival of about 15 months) of glioblastomas, it is almost impossible to cure these patients [2]. Moreover, the heterogeneous nature of glioblastomas makes it extremely challenging to develop an effective therapeutic approach with a uniform outcome for all patients [2,10].

Current standard glioblastoma treatment is multimodal in nature, involving surgery, radiotherapy, and chemotherapy. Surgery for glioblastoma aims for a maximal and safe resection of the tumor. Maximal resection not only helps to relieve the mass pressure in the brain but also prolong overall survival (OS) rate, as shown in a recent study by Yamaguchi et al. [11]. They reported that maximal resection for glioblastoma increases OS compared to incomplete resection [11,12,13,14]. Furthermore, the technological advancement of surgical therapy aided by fluorescence visualization with 5-aminolevulinic acid, the navigation-guided fence post procedure, and intraoperative MRI has facilitated maximal and almost complete resection of tumors [12,13,14]. After surgery, most patients undergo radiotherapy and chemotherapy concurrently. The current standard radiotherapy dosage regimen is 2 Gy per fraction per day for 5 days a week, continuously for 6 weeks, with a total dosage of 60 Gy [2]. Early radiotherapy soon after surgery has shown to increase progression-free survival (PFS). However, for OS no significant improvement has been shown [14]. Surgical and radiotherapeutic management of the disease has been extensively reviewed elsewhere [15,16,17,18,19,20,21,22].

Despite a moderate effect and controversial efficiency, chemotherapy has become a part of the standard treatment procedure for glioblastoma. Nowadays, the role of chemotherapy in the management of glioblastoma has become significant, with many studies dedicated to developing more efficient and effective chemotherapeutic treatments. Herewith, we review the current focus on therapeutic targets, how these targets are manipulated in chemotherapeutic development, and other novel therapeutic approaches for the treatment of glioblastoma.

## 2. Pathogenesis

Understanding the pathogenesis plays a key role not only in identifying disease biomarkers but also in designing and developing potential chemotherapeutic agents. Herein, we discuss the nine most promising signaling pathways that are involved in pathogenesis, and the possibility of targeting specific components of these pathways for the development of chemotherapeutic agents for glioblastoma.

### 2.1. IDH Mutation

**Figure 1:**
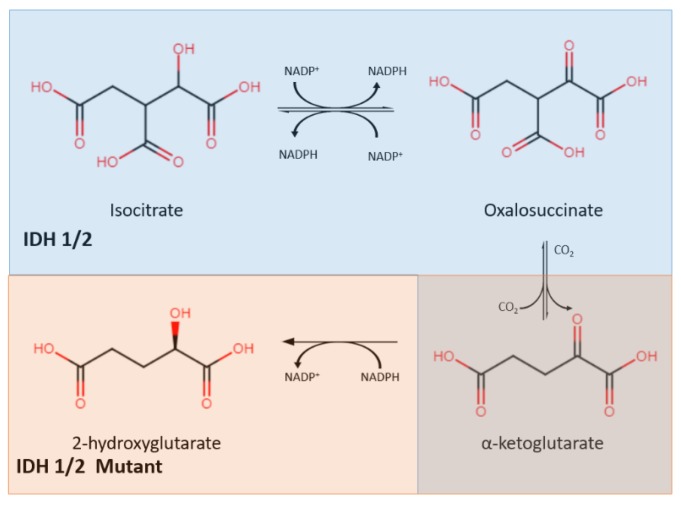
The IDH1/2 enzyme converts isocitrate into α-ketoglutarate (shown in the blue) while the mutant IDH1/2 enzyme converts α-ketoglutarate into 2-hydroxyglutarate (shown in orange). IDH: isocitrate dehydrogenase.

Isocitrate dehydrogenase (IDH) is an enzyme that plays a central role in the citric acid cycle. IDH has three isoforms: IDH1, IDH2, and IDH3. IDH1 is found in peroxisomes and the cytoplasm, while IDH2 and IDH3 are found in the mitochondrial matrix. Through oxidative decarboxylation by IDH1 and IDH2, isocitrate and NADP^+^ are converted to α-ketoglutarate (α-KG), NADPH, and carbon dioxide [23]. This takes place in a reversible, multistep process that starts with the oxidation of isocitrate to form oxalosuccinate, which is then decarboxylated to form α-KG, a cofactor for several enzymes (Figure 1) [24].

Mutations in IDH were found to be in almost all cases of secondary glioblastoma, as reported by Parsons et al. IDH mutations exist in high numbers in secondary glioblastomas and grade II and III gliomas but are rare in primary glioblastomas [25]. The IDH mutation involves both a loss and gain of regular enzymatic function [26]. It leads to a decrease in its binding affinity for isocitrate, preventing the conversion of isocitrate to α-KG. In addition, IDH mutation also increases its binding affinity for NADPH, which results in incomplete reaction by only reducing α-KG without carboxylation, forming 2-hydroxyglutarate (2-HG) instead of α-KG. The abnormal accumulation of 2-HG, an oncometabolite, is responsible for cancerogenesis [27]. This discovery resulted in mutant IDH (mIDH) inhibitors being identified as a new group of targeted cancer therapies which help to separate proliferating cancer cells. Popovici-Muller et al. reported that the mIDH1 inhibitor AGI-5198 was successful in 2-HG inhibition, and hindered the growth of mIDH1 glioma cells in vivo [28]. Optimization of AGI-5198 led to the finding of AG-120, which became the first mIDH1 inhibitor to achieve clinical proof-of-concept in human trials [28]. A selective R132H-IDH1 inhibitor, AG-5198, was discovered to almost completely block the ability of mIDH1 to produce 2-HG, and induced expression of genes involved in gliogenesis [29].

Results from clinical studies show that AG-221 (a selective inhibitor of mIDH2) has a promising inhibitory effect against advanced solid tumors [30]. There is an ongoing phase I clinical trial (NCT03343197) with AG-120 (mIDH1 inhibitor) and AG881 (non-specific IDH inhibitor). The objective of this trial is to understand the role of AG-120 and AG881 in the suppression of 2-HG by comparing the concentration of 2-HG in resected and treated tumors from IDH1 mutant glioma patients with the concentration of 2-HG in untreated tumor. Currently, two other chemotherapeutic agents, FT-2102 (a selective mIDH1 inhibitor) and IDH305 (an IDH1(R132H) inhibitor), are also in clinical trials (NCT03684811, NCT02381886). The objective of these clinical trials is to determine the dose-limiting toxicities (DLTs). More information on these clinical trials are available in Supplementary Table S1 (labeled with superscript 136, 148).

### 2.2. Notch Pathway

The Notch signaling plays an important role in cell differentiation, proliferation, and apoptotic events in different cell types and tissues, including neurons of the CNS. It is necessary to ensure that neural stem cells are promoted towards becoming glial cells instead of differentiating into another form [31]. Due to its key role in cell processes, it is easy for Notch signaling to deviate towards tumorigenesis.

There are four receptors involved in this pathway; Notch-1, Notch-2, Notch-3, and Notch-4. Notch-1 is found to be either a tumor suppressor or an oncogene based on the tissue type. Moreover, it has been found to be associated with glioma progression to determine the malignant phenotype of glioma. Notch-2, on the other hand, was identified as a prognostic marker for glioma along with Notch-3, which also promotes glioma cell proliferation. Lastly, Notch-4 was found to correlate with tumor aggressiveness [32].

Studies have shown the Notch pathway to be a potential and effective target in stem-like glioma cells, which were found to express Notch family genes [33]. In general, drugs inhibiting the Notch pathway are classified into three categories: α-secretase inhibitors, γ-secretase inhibitors, and other molecules. A detailed discussion of different classes of inhibitors and their biological effects has been published by Bazzoni et al. [34]. Ying et al. studied glioblastoma stem-like cell response to all-trans retinoic acid (RA) treatment. They found that RA can downregulate neurosphere cell expression of the Notch pathway targets Hes2, Hey1, and Hey2. When treated with RA, Notch receptor intracellular domain (NICD1) is forced to rescue glioblastoma neurospheres, thus causing inhibition of Hes2, Hey1, and Hey2. They concluded that this is an indication of RA affecting glioblastoma stem-like cells towards cell growth arrest, differentiation, and stem cell pool loss [35].

Similarly, Hovinga et al. performed a study on the relationship of neurosphere formation and CD133+ cells. It has been shown in the past that CD133+ cells are capable of self-renewal via the Notch pathway. Consequently, they discovered that Notch inhibition led to a decrease of neurosphere formation and CD133+ cells in glioblastoma while promoting an increased sensitivity to radiation [36]. Fan et al. studied glioblastoma neurosphere formation and Notch-2, which increases tumor cell growth. They demonstrated that inhibition of the Notch pathway, using gamma-secretase inhibitors, reduced glioblastoma neurosphere engraftment in vivo, which caused mice to live longer [33]. These studies indicate that inhibition of the Notch pathway is a potential therapeutic strategy to treat glioblastoma [33,36]. Currently there is one Notch inhibiting agent, CB-103, in a phase I/IIA clinical trial (NCT03422679) against metastatic solid tumors. The current primary outcome measures of the trial are to determine DLTs and antitumor efficacy.

### 2.3. Ceramide Signaling

**Figure 2:**
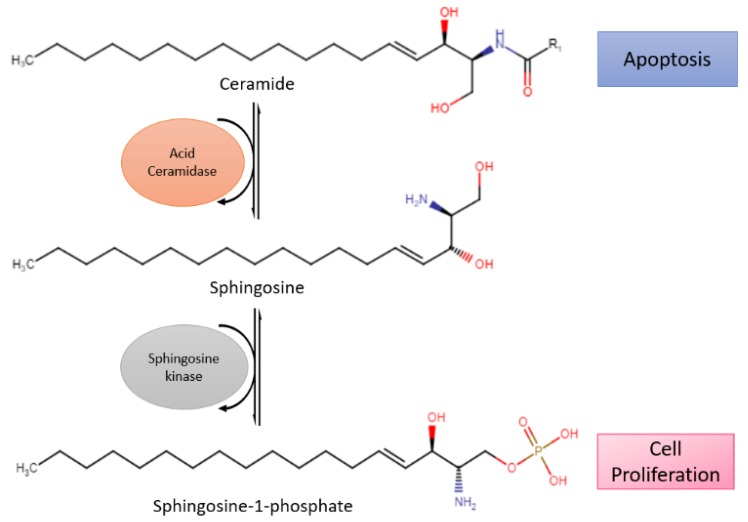
Reaction of acid ceramidase (ASAH1), a lysosomal enzyme that converts ceramides into sphingosine, which is further converted to sphingosine-1-phosphate (S1P) by sphingosine kinase. Ceramide promotes apoptosis while S1P stimulates cell survival and proliferation.

Acid ceramidase (ASAH1) is an enzyme that metabolizes ceramides into sphingosine and free fatty acids (Figure 2). Ceramides promote senescence and cell death [37]. On the contrary, sphingosine-1-phosphate (S1P), the immediate product due to metabolism, fosters cell survival and proliferation [38]. Histologically confirmed glioma cells have shown a change from ceramides to S1P, leading to higher S1P concentrations than ceramide. With lower amounts of ceramides, apoptosis occurs less, which allows the glioma cells to spread more freely [38]. In addition, modification of ASAH1 in glioblastoma enables it to be secreted to interstitial tissues, allowing it to transfer their malignant potential to nearby cells [39].

Previous studies have shown that glioblastomas express ASAH1 in high numbers. Doan et al. demonstrated that irradiated cell culture and tumor tissues have higher expression levels of ASAH1 compared to non-irradiated culture and tumor tissues, therefore leading to apoptotic resistance and glioblastoma recurrence [38]. This led to the identification of overexpression of ASAH1 as a potential biomarker associated with glioblastomas and the development of anticancer therapy. Although there are no drugs in clinical trials targeting ceramide signaling for glioblastomas, ASAH1 inhibitors (carmofur, N-oleoylethanolamine, and ARN14988) have been studied against multiple glioblastoma stem cell lines, U87, and patient-derived cell lines. In vitro studies of ASAH1 inhibitors have shown to be more effective against glioblastoma tumor cell lines compared to the Food and Drug Administration (FDA)-approved drug temozolomide (TMZ), therefore suggesting that ASAH1 inhibitors can restrain ASAH1 activity and increase tissue ceramide levels to induce apoptosis [40,41].

### 2.4. Vascular Endothelial Growth Factor (VEGF) Signaling Pathway

Vascular endothelial growth factor (VEGF), a potent angiogenic cytokine, stimulates the growth of new blood vessels to restore oxygen supply. The normal VEGF pathway starts when cells are lacking oxygen, which leads to the production of the hypoxia-inducible factor. This leads to releasing of VEGF followed by binding of the VEGF to VEGF receptors (VEGFRs), stimulating the tyrosine kinase pathway and ultimately resulting in angiogenesis. The normal signaling completes angiogenesis during embryonic development, collateral circulation, and following muscle injury and wounds [42].

Unfortunately, VEGF also plays a key role in promoting angiogenesis in glioma stem cells and optimizing the function and survival of its microenvironment. For survival of glioblastoma, a vascular supply must be maintained, and early extensions in the growing tumor receive this vascular supply by angiogenesis [43]. Hence, blocking the VEGF pathway and thereby inhibiting angiogenesis would be an effective strategy to treat the disease. Various anti-angiogenic agents have been shown to be effective in blocking the VEGF pathway, thereby treating several different cancers [44]. Though anti-VEGF therapy has been widely used and has shown benefits in the reduction of vasogenic edema associated with this disease, the overall survival benefit and resistance to therapy are yet to be improved. However, several approaches using combination therapy with radiotherapy, immunotherapy, cytotoxic drugs etc., in addition to anti-VEGF therapy showed improved results [45,46]. A recent study on combination therapy with platelet-derived growth factor (PDGF) inhibitors showed more promising results when combined with anti-VEGF therapy in terms of survival benefit and sensitization to therapy [47].

In the clinical setting, several receptor tyrosine kinase inhibitors (TKIs) such as tivozanib, cediranib, lenvatinib, sorafenib, sunitinib, and pazopanib are currently being studied for VEGFR inhibition. In addition, other therapeutic agents such as the TTAC-001 antibody, the VXM01 vaccine, and combination treatment with bevacizumab are also currently being studied. There are about 10 ongoing clinical trials and three recently published major clinical trials (Table 1) that are based on VEGF and VEGFR as the therapeutic targets for glioblastomas. The list of the ongoing trials is shown in Supplementary Table S1 (labeled with superscripts 59, 66, 93, 128, 160, 163, 186, 187, 197, and 219).

### 2.5. PDGF Signaling

Platelet-derived growth factor (PDGF) became a target for therapy for glioblastoma due to its ability to promote glioblastoma proliferation and survival [48]. In normal glial cells, PDGF signaling starts with the binding of the PDGF ligands such as PDGFA, PDGFB, and PDGFC to the platelet-derived growth factor receptor (PDGFRα or PDGFRβ). The PDGFR is classified as a cell surface receptor tyrosine kinase (RTK). Upon binding, the PDGFRs dimerize, allowing the subunits to cross phosphorylate several tyrosine residues in the receptor. This activated form acts as a docking site for multiple protein complexes to activate many signal transduction cascades, ultimately leading to DNA synthesis and cell proliferation [49,50].

On the contrary, a PDGF autocrine loop is exhibited in glioblastomas which should be absent in normal brain tissue [36]. Multiple observations have found PDGF overexpression in glioblastomas. PDGFA and PDGFB are highly expressed in comparison to the other ligands, with PDGFC being expressed the least [51]. Westermark noticed that the PDGFRα gene is amplified, mutated, or rearranged in glioblastoma tumors, playing a role in oncogenesis [52]. Similarly, Shih et al. found PDGF and PDGFR to be overexpressed in glial tumor cell lines and samples correlating with higher tumor grade. Autocrine signaling in tumor proliferation was tested in cell culture where PDGF inhibitors were able to limit colony activity and cell growth [49]. Popescu et al. investigated a PDGFR inhibitor, AG1433, and discovered that both the growth factor and its receptors can control cell proliferation, differentiation, and apoptosis in glioblastoma. They remarked that it was able to reduce cell survival to 56.5% with the highest concentration (100 μM) at 72 h [53]. Another study by Hong et al. found the TKI imatinib to be successful at enhancing the radiosensitivity and chemosensitivity of gliomas. Moreover, it has been observed that it can radiosensitize the cells and inhibit tyrosine phosphorylation of numerous intracellular proteins in a dose-dependent manner [54]. Another PDGFRα inhibition study conducted by Mangiola et al. found a significant decrease in cell proliferation in core cancer stem cells, by about 38 ± 9.5%. They also observed a decrease in the modulation of PDGFRα expression [55]. These studies indicate that PDGF is a well-studied pathway that could lead to possible treatments for glioblastoma.

In clinical settings, several TKIs such as tandutinib, crenolanib, sorafenib, sunitinib, and pazopanib are currently being studied. There are about six ongoing clinical trials and two recently published major clinical trials (Table 1) that are based on PDGF and PDGFR as the therapeutic targets for glioblastoma. The current ongoing trials are listed in Supplementary Table S1 (labeled with superscripts 104, 128, 163,186,197, and 219).

### 2.6. Epidermal Growth Factor Receptor (EGFR) Pathway

The epidermal growth factor receptor (EGFR) is a transmembrane cell RTK that binds extracellular signaling ligands such as epidermal growth factors and transforming growth factor-α to its extracellular domain. In normal glial cells, the EGFR pathway starts when the receptor binds to its signaling ligand and becomes activated, undergoing transitions to an active homodimer from an inactive monomer. This dimerization induces intracellular protein-tyrosine kinase activity and results in tyrosine residues being autophosphorylated in the C-terminal domain of EGFR. Such autophosphorylation stimulates the initiation of many signal transduction cascades, which ultimately lead to DNA synthesis, cell proliferation, migration, and adhesion [56].

Mutations in EGFR have been widely recognized to be involved in the pathogenesis of glioblastomas. The amplification of EGFR was found to be more commonly present in primary glioblastomas (40%), and rarely present in secondary glioblastomas [57]. Furthermore, EGFR amplification was found to be rare or nonexistent in pediatric glioblastomas [58]. In a population-based study conducted by Ohgaki et al., EGFR amplification was found to be detected only in glioblastoma patients older than 35 years, confirming the results of the previous study [59]. For tumors with amplified EGFR expression, about half of those cases have the EGFRvIII variant, which is an ideal target for therapies [60,61].

Though EGFR was one of the first molecule linked to oncogenesis of glioblastoma, targeting it has been challenging in this disease. Hence, recent studies have focused on both immunotherapy as well as tyrosine kinase inhibitors (TKIs). For example, OSI-774, an EGFR-TKI, has shown to be promising in a study conducted by Halatsch et al. They showed that it induces apoptosis in malignant glioblastoma and is a promising agent against secondary glioblastoma [62]. However, phase I/II clinical trials of another TKI, lapatinib, showed limited antitumor activity in patients. Though TKIs are promising, EGFR inhibitors in the pre-clinical settings as well as drug delivery and activity must be evaluated further [63]. EGFR-targeting therapeutic agents such as dacomitinib, nimotuzumab, ABBV-321, AMG596, CART-EGFRvIII T cells, EGFR(v)-EDV-DOX, axitinib, cabozantinib, neratinib, afatinib, alectinib, and tesevatinib are currently in clinical studies. From January 2017 to September 2019, about six major clinical trials were published (Table 1), while currently there are about 19 ongoing clinical trials based on EGFR-targeting therapeutic agents and tyrosine kinase inhibition for glioblastomas. A list of ongoing clinical trials is provided in Supplementary Table S1 (labeled with superscripts 5, 36, 38, 80, 88, 99,122, 124, 125, 137, 157, 158, 207 for EGFR-targeting therapeutic agents and 8, 21, 28, 41, 81, 163 for TKIs).

### 2.7. PI3K/AKT/mTOR Pathway

**Figure 3:**
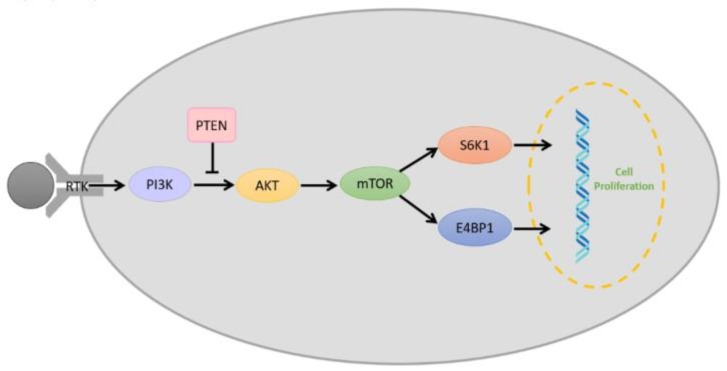
Schematic representation of a simplified overview on the PI3K/AKT/mTOR pathway with the role of phosphate and tensin homolog (PTEN) and receptor tyrosine kinase (RTK).

The PI3K/AKT/mTOR pathway (Figure 3) is a vital intracellular signaling pathway for regulating the cell cycle. Phosphatidylinositol 3-kinases (PI3Ks) are intracellular signal transducer enzymes that can activate serine/threonine-specific protein kinase (AKT) through phosphorylation. Subsequently, AKT can activate the mammalian target of rapamycin (mTOR). mTOR forms two complexes which are characterized by different binding partners; mTOR complex 1 (mTORC1) and mTOR complex 2 (mTORC2) [64]. mTORC1 is rapamycin-sensitive and is activated by at least five cues (growth factors, stress, energy status, oxygen, and amino acid concentration), and promotes glial cell growth upon activation by eukaryotic translation initiation factor 4E binding protein 1(E4BP1) and ribosomal protein S6 kinase (S6K) [64,65]. Conversely, mTORC2 is insensitive to rapamycin, which drives the glial cell proliferation, motility, and survival through the activation of AGC protein kinases [64,65,66]. However, it is found that overactivation of the PI3K/AKT/mTOR pathway reduces in the survival of glioblastoma patients and increases in the aggression of the tumor as it overstimulates processes responsible for cell proliferation, survival and migration in glioblastoma [67,68]. Therefore, researchers have identified PI3K, AKT, and mTOR as molecular targets for glioblastomas.

Recently, a few preclinical trials have found mTOR inhibitors to be successful. For example, Mecca et al. found that CC214-1 and CC214-2, mTOR kinase inhibitors, were capable of inhibiting glioblastoma growth by blocking mTOR2C2 activity both in vitro and in vivo [65]. In the clinical setting, PI3K inhibitors such as BKM120, regorafenib, GDC-0084, and fimepinostat as well as mTOR inhibitors such as temsirolimus, everolimus, CC-115, ABI-009, AZD2014, sapanisertib, and siroquine are currently being studied. From January 2017 to September 2019, about four major clinical trials were published (Table 1), while currently, there are about 11 ongoing clinical trials based on P13K and mTOR inhibition for glioblastomas. The list of ongoing clinical trials is provided in Supplementary Table S1 (labeled with superscripts 72, 133, 138 for P13K inhibitors and 7, 9, 33, 63, 90, 126, 202, 205 for mTOR inhibitors).

### 2.8. Phosphate and Tensin Homolog (PTEN) Signaling

Another key element associated with glioblastoma in the PI3K pathway is phosphate and tensin homolog (PTEN). PTEN is a tumor suppressor that antagonizes PI3K signaling and prevents AKT activation via its lipid phosphatase activity (Figure 3) [69]. In glioblastomas, it has been reported that PTEN is inactivated due to mutations. A single mutation in one of the homolog genes is insufficient to initiate tumor growth; however, the deletion of one or both results in uncontrollable cell growth [70]. It has also been found that PTEN can sensitize glioma cells to chemotherapy and radiation therapy [71], hence making PTEN a molecular target for glioblastoma immunotherapy.

Recent developments in the preclinical setting have focused on correction to PTEN mutation. A study reported the correction of PTEN in glioblastoma using the adeno-associated virus-mediated gene that reduced the cellular proliferation in the glioblastoma cell lines, indicating that it could be a potential treatment for this disease [72]. Furthermore, another study illustrated that correction of the mutant allele of PTEN in glioblastoma cells lines (42MGBA and T98G) using gene editing resulted in reduced cell proliferation [73].

### 2.9. SHH Signaling

In normal glial cells, signaling starts with the sonic hedgehog (SHH) glycoprotein binding to and inactivating the protein Patched1 and co-receptors, leading to inactivation of the protein Smoothened (SMO). However, when SMO is activated, the nuclear localization of glioma-associated (GLI) transcription factors takes place. Once GLI enters the nucleus it leads to the activation of GLI1 and GLI2 transcription factors. Such activation promotes target activation in SHH signaling, leading to proliferation, angiogenesis, epithelial-to-mesenchymal transition, and stem cell self-renewal [74,75]. In glioblastomas, the abnormal activation of SHH signaling typically by mutation in Patched1 and/or activating mutations in SMO leads to the transformation of adult stem cells into glioblastoma stem cells.

Therefore, SHH signaling has become one of the focal points for glioblastoma treatment since mutations in the pathway play a key role in cell proliferation and tumorigenesis. Since SMO inhibition prevents downstream activation of GLI, SMO is an important molecular target for the development of SHH pathway inhibitors [76]. SMO inhibitors such as vismodegib, trametinib, and glasdegib have been under investigation for glioblastoma [77]. Currently, there are three ongoing clinical trials based on SMO inhibition for glioblastomas. These are listed in Supplementary Table S1 (labeled with superscripts 34, 131, and 139).

## 3. Current Chemotherapeutic Development

Identifying the molecular targets of glioblastomas and understanding pathogenesis is one part of the puzzle, and the next is developing the chemotherapeutic agents. Therefore, herewith we discuss the current FDA-approved chemotherapeutic agents (Table 2), as well as recently published (Table 1) and ongoing (Supplementary Table S1) drug candidates that are in the pipeline at different stages of clinical development.

### 3.1. FDA-Approved Chemotherapeutic Agents

Currently, three chemotherapeutic agents (TMZ, bevacizumab, and carmustine) are available to patients with glioblastoma [78,79]. Results from randomized clinical study in 573 patients demonstrate that the addition of TMZ to radiotherapy significantly increases OS (27.2% vs. 10.9% in radiotherapy alone at 2 years). The same study found that O^6^-methylguanine-DNA methyl-transferase (MGMT) gene methylation is a positive prognostic indicator for TMZ chemotherapy for newly diagnosed patients [80]. On the other hand, bevacizumab is an anti-VEGF monoclonal antibody (refer to Section 2.4. for pathogenesis) that has been approved by the FDA for the treatment of recurrent glioblastoma. It has been clinically observed that bevacizumab has anti-glioma activity with improvement in PFS; however, it has no significant activity in terms of OS [81]. A clinical trial on newly diagnosed glioblastoma patients with bevacizumab has shown to have no significant activity in terms of OS but longer PFS compared to the placebo group (10.7 months vs. 7.3 months) [82]. Carmustine, a nitrosourea compound which is used in the treatment of the disease, is now avoided due to clear demonstration of severe bone marrow, liver and kidney toxicity [2]. However, local delivery of carmustine in the form of an implant in the resection cavity followed by surgery can reduce systemic adverse events, and can improve median survival of the patients both in recurrent and newly diagnosed glioblastoma [83]. The required doses and dosage regimens of the chemotherapeutic agents are summarized in Table 2.

### 3.2. Published Clinical Trials

Currently, several drug candidates are in the pipeline at different stages of clinical development. Table 1 lists the data of 62 clinical trials of some major drugs and biologicals published from January 2017 to December 2019, revealing both encouraging and not-so-encouraging outcomes.

### 3.3. Ongoing Clinical Trials

Currently, there are several ongoing clinical trials of various candidates at different stages of clinical development. Supplementary Table S1 includes a comprehensive list of therapeutic agents, the mechanism of action, clinical trial phase, estimated completion date, and the clinical trial identifier for 286 ongoing clinical trials summarized from www.clinicaltrials.gov.

## 4. Novel Therapies

A common setback with chemotherapy is that it induces severe side effects such as nausea, vomiting, hair loss, and a weakened immune system. Therefore, studies have been conducted to look for alternate therapies. Listed below are few novel therapies that have been emerging for the treatment of glioblastoma. 

### 4.1. Laser Interstitial Thermal Therapy (LITT)

When surgical removal of a tumor is unsuitable, LITT offers treatment in glioblastoma patients by destroying the tumor cells with localized elevated temperature [145]. Thermal therapy can also be achieved using radiofrequency, ultrasound, microwave, and magnetic nanoparticle (MNP) treatments [146]. However, laser-induced thermotherapy offers the advantage of minimal invasiveness. Studies have found that MRI-guided LITT is safe [145] and can also disrupt peritumoral blood–brain barrier (BBB) for therapeutic permeability [147]; however, it should be used with caution. Most patients can be discharged within 24 h of post operation [148]. A study of a small group of patients has observed the efficacy of LITT in recurrent glioblastoma as an alternative to surgery [149]. Retrospective analysis also found that LITT enhances the PFS of difficult-to-access high-grade gliomas [150]. However, comprehensive studies are needed to be performed to establish LITT as a substitute to standard surgical removal of the tumor. Currently, there are several clinical trials (NCT02880410, NCT03022578) ongoing both in newly diagnosed and recurrent glioblastoma as well as in combination with chemotherapy (NCT03341806, NCT03277638).

### 4.2. Tumor Treating Fields (TTFields)

TTFields is a technology which creates alternating electric fields of low-intensity (1–3 V/cm) and intermediate frequency (100–300 KHz), interrupting the prolific cell division of cancerous cells and leaving the quiescent and non-dividing cells in the human body unaffected [151]. Optune^®^, a device made by Novocure, is the commercial example of TTFields. It was approved by the FDA for the treatment of recurrent and newly diagnosed, supratentorial, and histologically confirmed glioblastomas in 2011 and 2015, respectively. For recurrent glioblastomas, it is intended to be used as monotherapy while for newly diagnosed glioblastomas it is used along with adjuvant chemotherapy [81,152,153]. The device is patient-operated and mounted on the shaved scalp with the support of an insulated transducer array [152]. Results from randomized clinical trials demonstrate that incorporation of TTFields along with adjuvant TMZ chemotherapy significantly increases OS (20.9 months vs. 16.0 months) and PFS (6.7 months vs. 4.0 months) without any serious negative impact other than itchy skin with respect to patient health-related quality of life [154,155]. With fewer side effects, TTFields is likely to be of benefit to patients; however, its use is limited because of the high cost of the technology [152]. Currently, there are several ongoing clinical trials (NCT01925573, NCT03780569) in combination with chemotherapy for new and recurrent glioblastomas.

### 4.3. Immunotherapy

Immunotherapy selectively targets and kills tumor cells. There are numerous strategies towards the development of an immune response such as immune checkpoint inhibitors (CPIs), oncolytic virus therapy, vaccine therapies, modified T cell therapies, and gene therapies [156,157]. These are cell-based and non-cell-based therapies which are categorized as active or passive immunotherapy, grounded on their mechanism of actions [157]. Discussed below are the different types of immunotherapy.

#### 4.3.1. Immune Checkpoint Inhibitors

Inspired from the results in treating other cancers and with the capability to cross BBB, CPIs such as nivolumab, pembrolizumab, durvalumab, atezolizumab, and pidilizumab have been under investigation against recurrent glioblastomas. Although the results from preliminary clinical trials are not very exciting, significant efforts are ongoing to develop CPIs both as monotherapy and combination therapy [158,159].

#### 4.3.2. T-Cell Therapy

T cell therapy has been demonstrated as a promising and emerging therapeutic strategy against glioblastomas, where T cells are engineered to express chimeric antigen receptors (CARs). Unlike hematologic malignancies, there is no FDA-approved T cell therapy for glioblastoma. Recent studies on CAR T cells have been focused on targeting EphA2, EGFR, CD70, HER2, and IL-13Rα (Interleukin-13 receptor α) [158,160]. Because of extensive tumor heterogeneity, T cell therapy is intended as a combination therapy instead of a single therapy for the treatment of glioblastomas [158].

#### 4.3.3. Viral Therapy

This is considered the part of immunotherapy in which an immunogenic oncolytic virus exerts its effect in a variety of mechanisms which include direct oncolysis, virus-induced anti-tumor immunity, immunoregulatory inserts, etc. [161]. Due to the highly immunosuppressive nature of the glioblastoma tumor, the immunostimulatory effect of oncolytic viruses has become the concentration of current design of viral therapy [158]. Results from clinical trials demonstrate that the combination treatments of viral therapy with immunotherapy, radiotherapy, or chemotherapy result in better patient outcomes. Although there is currently no FDA-approved viral construct for the treatment of glioblastomas, many studies are ongoing at different stages of clinical trials, both as monotherapy and multimodal therapy. Eleven of the current ongoing clinical trials based on viral therapy are mentioned in Supplementary Table S1 (labeled with superscripts 12, 13, 14, 56, 79, 87, 103, 116, 117, 145, and 179). The current status of oncolytic viral therapy has been reviewed by Martikainen and Essand [161].

#### 4.3.4. Vaccine Therapy

Vaccines in glioblastoma are not preventive but considered as a form of active immunotherapy that can stimulate and adapt immune responses against tumor-associated antigens [162]. They are cell-based, for example patient-derived dendritic cells and autologous tumor cell vaccines, and/or non cell-based, for example peptide and heat shock protein vaccines. Peptide vaccines are specifically engineered peptide sequences that induce targeted immunity against major histocompatibility complex bound tumor associated antigens. They are co-administered with an immunostimulant adjuvant for antigen cross-presentation. Other versions of the vaccine therapy are heat shock protein vaccines, which are designed to create a highly specific antitumor inflammatory response. Autologous tumor cell vaccines are a technique where cytotoxic T lymphocytes are induced in patient-derived tumor cells and reintroduced to the patient in order to create an antitumor immune response [163]. Dendritic cell vaccines are the final variation of vaccine therapy. Dendritic cells are antigen-representing cells that are extracted from the patient, cultured, loaded with glioma cell antigens, and reintroduced to the patient, thus activating the CD8+ and CD4+ T cells, resulting in tumor cell death [156]. Vaccines as immunotherapy provide high specificity and low toxicity. Some of the current ongoing clinical trials based on vaccine therapy are provided in Supplementary Table S1 (labeled with superscripts 105, 113, 114, 119, 140, 144, and 224). The summary of recent studies from clinical trials can be found in the corresponding references [158,162,164].

## 5. Conclusions

Tumor heterogeneity, patient-to-patient variability, and different stages of disease progression at the time of diagnosis foster complexity in the treatment of glioblastoma. While there are a few FDA-approved multimodal-approach treatments for glioblastoma, survival is still poor in the majority of the patients. As discussed in this manuscript, exploration for understanding the molecular-level information on the mechanistics of neoplasms has led to the design of multiple new compounds which are now under investigation at different stages of clinical development. Based on the ongoing clinical trials discussed here, new treatment options are likely to evolve in coming years. In addition, extensive research is ongoing to develop other novel strategies to better combat the disease. Ultimately, the overall goal is to lessen patient suffering by providing a better standard of life and increasing overall survival.

## Figures and Tables

**Table 1:** Published major clinical trials of targeted therapies from January 2017 to December 2019.

Treatment	Disease Type	Clinical Trial Phase	No. of Patients	Result (s)	Reference
^1^ Alisertib + RT	Recurrent	I	17	OS-6: 88.2%; Median survival: 11.1 months; PFS-6: 35.5%.	[84]
^2^ Lomustine + ^52^ TMZ vs. ^52^TMZ	Primary	III	141	Median OS: 48.1 months (32.6—not assessable) vs. 31.4 months (95% CI, 27.7—47.1); AEs: 59% vs. 51% of patients.	[85]
^3^ Disulfiram + copper	Recurrent	II	21	ORR: 0%; Clinical benefit: 14%; Median PFS: 1.7 months; Median OS: 7.1 months; DLTs: 4%.	[86]
^4^ Ortataxel	Recurrent	II	40	PFS-6: 11.4%; AEs: Neutropenia and hepatotoxicity (13.2%) and leukopenia (15.8%).	[87]
^5^ Buparlisib	Recurrent	II	15+50	Reduction of phosphorylated AKT: 42.8%; PFS-6: 8%; Median PFS: 1.7 months (95% CI, 1.4 to 1.8 months); AEs: Lipase elevation (10.8%), fatigue (6.2%), hyperglycemia (4.6%), elevated ALT (4.6%).	[88]
^6^ Regorafenib vs. ^2^ Lomustine	Recurrent	II	119	Patients died at cut-off: 71% vs 95%; Median OS: 7.4 months (95% CI, 5.8—12.0) vs. 5.6 months (95% CI, 4.7–7.3); AEs: 56% (hand–foot skin reaction, increased lipase, blood bilirubin) vs. 40% (decreased platelet count, decreased lymphocyte count, neutropenia).	[89]
^52^ TMZ + RT → ^52^ TMZ + ^7^ irinotecan (CPT-11)	Primary	II	152	Median OS: 16.9 months vs 13. 7 months (*p* = 0.03) in historical control; Grade 3/4 hematologic toxicity: 38% vs. 14% in Stupp trial.	[90]
^8^ Valproic acid + ^52^ TMZ + RT	Primary	II	6	Late toxicity in long-term survivors: neurological, pain, and blood/bone marrow toxicity (mostly grade 1/2).	[91]
^52^ TMZ + ^9^ memantine + ^10^ mefloquine + ^11^ metformin (adjuvant)	Primary	I	81	DLTs: Dizziness (memantine), gastrointestinal effects (metformin); AEs: Lymphopenia (66%); Median survival: 21 months; 2-year survival: 43%; MTDs (doublet, triplet, quadruplet): Memantine (20 mg b.i.d., 10 mg b.i.d., 10 mg b.i.d.), mefloquine (250 mg 3 times weekly, 250 mg 3 times weekly, 250 mg 3 times weekly), metformin (850 mg b.i.d., 850 mg b.i.d., 500 mg b.i.d.).	[92]
RT + ^52^ TMZ + ^12^ bevacizumab (BEV) → ^2^ CCNU + ^12^ BEV/ ^2^ CCNU + placebo → ^12^ BEV/placebo + chemotherapy	Recurrent	II	296	No survival benefit and no safety concerns.	[93]
^13^ ERC1671 + ^12^ bevacizumab vs. ^12^ bevacizumab + placebo	Recurrent	II	9	Median OS: 12 months vs. 7.5 months.	[94]
^14^ Palbociclib (with and without resection)	Recurrent	II	22	Median PFS: 5.14 weeks (5 days–142 weeks); Median OS: 15.4 weeks (2–274 weeks).	[95]
^15^ Iniparib + RT + ^52^ TMZ	Primary	II	81	Median OS: 22 months (95% CI, 17-24); 2- and 3-year survival: 38% and 25%; Grade 3 AEs: 27% of patients.	[96]
^16^ Depatuxizumab mafodotin + ^52^ TMZ	Recurrent	I	60	AEs: blurred vision (63%), fatigue (38%), and photophobia (35%); Grade 3/4 AEs: Ocular (22%), non-ocular (22%); ORR: 14.3%; PFS-6: 25.2%; OS-6: 69.1%.	[97]
^17^ Fotemustine (120 or 140 mg/m)	Recurrent	I/II	37	Toxicity: Grade 3 and 4 thrombocytopenia (4 of 6 patients at 140 mg/m vs. 3 of 31 patients at 120 mg/m); Median PFS: 12.1 (1–40.2) weeks; OS: 19.7 (1–102) weeks.	[98]
^18^ AZD1775	Recurrent	0	20	BBB permeability; Median unbound tumor to plasma concentration ratio: 3.2.	[99]
^19^ Bortezomib + ^52^ TMZ + RT	Primary	II	24	Median PFS: 6.2 months (95% CI 3.7–8.8); Median OS: 19.1 months (95% CI, 6.7–31.4); no unexpected AEs.	[100]
^20^ Carboxyam- idotriazole orotate + ^52^TMZ	Recurrent/ Primary	Ib	47	DLTs: none; Recommended phase II dose: 600 mg/day.	[101]
^12^ Bevacizumab + RT vs. RT	Primary	II	75	Median PFS: 7.6 vs. 4.8 months, *p* = 0.003; OS: 12.1 vs. 12.2 months, *p* = 0.77.	[102]
^21^ Interferon β + ^52^ TMZ + RT vs. ^52^TMZ + RT	Primary	II	122	OS: 24.0 vs. 20.3 months; Median PFS: 8.5 vs. 10.1 months; Neutropenia: 20.7 vs. 12.7 % (concomitant) and 9.3% vs. 3.6% (maintenance).	[103]
^12^ Bevacizumab + ^52^ TMZ	Primary	II	66	Median OS: 23.9 weeks (95% CI 19–27.6); Median PFS: 15.3 weeks (95% CI, 12.9–19.3); AEs: Grade ≥ 3 hematological events (20%), high blood pressure (24%), venous thromboembolism (4.5%), cerebral hemorrhage (3%), and Intestinal perforation (3%).	[104]
^3^ Disulfiram (with or without copper) + adjuvant ^52^ TMZ	Primary	I	18	MTD: Disulfiram 500 mg daily was well tolerated, 1000 mg daily was not; Median PFS: 4.5 months (95% CI 0.8–8.2); Median OS: 14.0 months (95% CI 8.3–19.6).	[7]
^22^ Temsirolimus + ^23^ sorafenib	Recurrent	I/II	41	MTD (Phase I): sorafenib (200 mg twice daily) and Temsirolimus (20 mg weekly); Median PFS and OS (Phase II): 2.6 months vs. 1.9 months (VEGF inhibitor-naïve vs. prior VEGF inhibitor patients) and 6.3 months vs. 3.9 months (VEGF inhibitor-naïve vs. prior VEGF inhibitor patients).	[105]
^24^ Trebananib vs. ^24^ trebananib + ^12^ bevacizumab	Recurrent	II	48	Trebananib: Well tolerated as monotherapy; Trebananib + Bevacizumab: PFS-6 (24.3%, 95% CI, 12.1%-38.8%), Median OS (9.5 months, 95% CI, 7.5–4.7 months), OS-12 (37.8%, 95% CI, 22.6%–53.0%).	[106]
^25^ Vorinostat + ^12^ bevacizumab + ^52^ TMZ	Recurrent	I/II	9+39	MTD (phase I): 400 mg for vorinostat; PFS-6 (phase II): 53.8% (95% CI, 37.2–67.9).	[107]
^25^ Vorinostat + ^12^ bevacizumab	Recurrent	II	40	PFS-6: 30.0% (95% CI, 16.8%–44.4%); Median OS: 10.4 months (95% CI, 7.6–12.8 months); AEs (grade 2): Lymphopenia (55%), leukopenia (45%), neutropenia (35%), and hypertension (33%). AEs (grade 4): Leukopenia (3%), neutropenia (3%), sinus bradycardia (3%), and venous thromboembolism (3%).	[108]
^26^ Everolimus + RT + ^52^ TMZ vs. RT + ^52^TMZ	Primary	II	171	Median PFS: 8.2 vs. 10.2 months, *p* = 0.79); Median OS: 16.5 vs. 21.2 months, *p* = 0.008)	[109]
^27^ AXL1717	Recurrent	I	9	Tumor response: 44%; AEs: Neutropenia.	[110]
^28^ ONC201	Recurrent	II	17	Median OS: 41.6 weeks; PFS-6: 11.8%; Drug-related serious AEs: None; Plasma pharmacokinetics (2-h post-dose): 2.6 µg/mL.	[111]
^29^ Nivolumab (with or without ^30^ ipilimumab)	Recurrent	I	40	Nivolumab monotherapy better tolerated; AEs: fatigue, and diarrhea; Tumor-cell programmed death ligand-1 expression ≥1% (68%).	[112]
^31^ Cabozantinib	Recurrent	II	70	ORR: 4.3%; Median duration of response: 4.2 months; PFS-6: 8.5%; Median PFS: 2.3 months; Median OS: 4.6 months. AEs: Fatigue, diarrhea, increased alanine aminotransferase, headache, hypertension, and nausea. 48.6% resulted in dose reductions (140 mg/day to 100 mg/day).	[113]
^31^ Cabozantinib (140 mg/day vs. 100 mg/day)	Recurrent	II	152	ORR: 17.6% vs. 14.5%; PFS-6: 22.3% vs. 27.8%; Median PFS: 3.7 months in both; Median OS: 7.7 vs. 10.4 months; AEs (grade 3/4): 79.4% vs. 84.7%; Dose reduction due to AEs: 61.8% vs. 72.0%.	[114]
^25^ Vorinostat + ^52^ TMZ + RT	Primary	I/II	15+107	MTD: 300 mg/day; DLTs: Grade 4 neutropenia and thrombocytopenia and grade 3 aspartate aminotransferase elevation, hyperglycemia, fatigue, and wound dehiscence; Phase II OS-15 months: 55.1% (median OS 16.1 month); Phase II toxicities: Lymphopenia (32.7%), thrombocytopenia (28.0%), and neutropenia (21.5%).	[115]
^23^ Sorafenib + ^32^ tipifarnib	Recurrent	I	24	Study stopped because of excessive toxicities. Last dose reached: 200 mg and 100 mg twice a day for sorafenib and tipifarnib, respectively.	[116]
^33^ Axitinib vs. ^33^ axitinib + ^2^ lomustine	Recurrent	II	79	ORR: 28% vs. 38%; PFS-6: 26% (95% CI, 14–38) vs. 17% (95% CI, 2–32); Median OS: 29 weeks (95% CI, 20–38) vs. 27.4 weeks (95% CI 18.4–36.5); Toxicities: Grade ¾ neutropenia (0 vs. 21%) and thrombocytopenia (4 vs. 29%).	[117]
^34^ Rindopepimut + ^52^ TMZ vs. ^52^ TMZ	Primary	III	745	OS for patients with MRD: 20.1 months (95% CI, 18.5–22.1) vs. 20.0 months (18.1–21.9); Grade 3/4 AEs: Thrombocytopenia (9% vs. 6%), fatigue (2% vs. 5%), brain edema (2% vs. 3%), seizure (2% vs. 2%), and headache (2% vs. 3%); Mortality by AEs: 4% vs. 3%.	[118]
^12^ Bevacizumab + ^52^ TMZ	Recurrent	II	30	ORR: 51 weeks; PFS-6: 52%; Median time to tumor progression: 5.5 months.	[119]
^52^ TMZ (150–200 mg/m^2^/day) + RT (60 Gy in 5 days)	Primary	II	35	OS: 22 months; Hematologic toxicities: ≤grade 2.	[120]
^35^ Lapatinib + ^52^TMZ + RT	Primary	II	12	Higher dose correlates to lymphopenia; Common AEs: fatigue, rashes, and diarrhea	[121]
^36^ Dacomitinib	Recurrent	II	30 + 19	PFS-6: 10.6%; Median PFS: 2.7 months; Median OS: 7.4; Best overall response: 4.1%; Common AEs: Diarrhea and rash; Drug-related AEs: 40.8% (grade 3/4).	[122]
^37^ HER2-CAR VSTs (HER2 specific CAR-modified virus-specific T cells)	Recurrent	I	17+7	No dose-limiting toxic effects; Presence in peripheral blood: up to 12 months; Stable disease: 7 out of 16 patients for 8 weeks to 29 months; Disease progression: 8 out of 16 patients; Median OS: 11.1 months (95% CI, 4.1–27.2 months) after infusion.	[123]
^38^ Irinotecan liposome injection (nal-IRI)	Recurrent	I	16 + 18	MTD: 120 mg/m^2^ (WT cohort), 150 mg/m^2^ (HT cohort); DLTs: Diarrhea, dehydration and/or fatigue.	[124]
^39^ C_p_GODN→RT + ^52^ TMZ vs. RT + ^52^ TMZ	Primary	II	81	2 years OS: 31% vs. 26%; Median PFS: 9 vs. 8.5 months.	[125]
^40^ Aflibercept + RT + ^52^ TMZ→ ^52^ TMZ	Primary	I	59	MTD: 4 mg/kg for 2 weeks; DLTs: G3 deep vein thrombosis, G4 neutropenia, G4 biopsy-confirmed thrombotic microangiopathy, G3 rash, G4 thrombocytopenia; Treatment discontinuation: disease progression (47%), toxicities (36%), others (14%), full course (3%).	[126]
^41^ Onartuzumab + ^12^ bevacizumab vs. placebo + ^12^ bevacizumab	Recurrent	II	129	Median PFS: 3.9 vs. 2.9 months; Median OS: 8.8 vs. 12.6 months; AEs (G ≥ 3): 38.5% vs. 35.9%.	[127]
^42^ Tivozanib	Recurrent	II	10	Progressive disease: 80%; Median PFS: 2.3 months; Median OS: 8.1 months.	[128]
^43^ MEDI-575	Recurrent	II	56	PFS-6: 15.4% (90% CI 8.1–24.9 months); Stable disease: 41.1%; Median PFS: 1.4 months (90% CI 1.4–1.8); Median OS: 9.7 months (90% CI, 6.5–11.8); Treatment-related AEs: Diarrhea (16%), nausea (13%), and fatigue (13%).	[129]
^44^ Bortezomib + ^52^ TMZ + ^12^ bevacizumab	Recurrent	I	12	MTD: 75 mg/m^2^ for TMZ; PFS: 3.27 months: Mean OS: 20.75 months.	[130]
^45^ Nimustine + ^52^ TMZ	Recurrent	I/II	15 + 40	MTD: TMZ (150 mg/m^2^), nimustine (40 mg/m^2^); ORS: 11%; Stable disease: 68%; PFS-6 and PFS-12: 24% (95% CI, 12–35%) and 8% (95% CI, 4–15%); Median PFS: 13 months (95% CI, 9.2–17.2 months); OS-6 and OS-12: 78% (95% CI, 67–89%) and 49% (95% CI, 33–57%); Median OS: 11.8 months (95% CI, 8.2–14.5 months).	[131]
^46^ Tandutinib	Recurrent	I/II	19+30	MTD: 600 mg twice daily; Phase II terminated as PFS-6 not achieved.	[132]
^47^ Imatinib + RT vs. ^47^ imatinib + re-irradation	Recurrent	II	51	Median OS: 5.0 months (95% CI, 0-24.1 months) vs. 6.5 months (95% CI 0–32.5 months; Median PFS: 2.8 months (95% CI 0–8.7 months) vs. 2.1 months (95% CI 0–11.8 months).	[133]
^48^ BKM120 + ^12^ bevacizumab	Recurrent	I/II	88	MTD: 60 mg PO (orally) daily; PFS-6: 36.5%; ORR: 26%; TRTs: 57%.	[134]
^49^ Perifosine	Recurrent	II	30	PFS-6: 0%; PFS: 1.58 months (95% CI, 1.08–1.84 months); Median OS: 3.68 months (95% CI, 2.50–7.79 months).	[135]
^50^ Dovitinib (naïve vs. progressed on prior antiangiogenic therapy)	Recurrent	II	19+14	PFS-6: 12% vs. 0%; TTP: median 1.8 months vs. 0.7–1.8 months.	[136]
^51^ Nimotuzumab + ^52^ TMZ + RT	Primary	II	39	ORR: 72.2%; Median OS: 24.5 months; Median PFS: 11.9 months.	[137]
^53^ Ponatinib	Recurrent	II	15	PFS-3: 0; Median PFS: 28 days (95% CI, 27–30); Median OS: 98 days (95% CI 56–257).	[138]
^2^ Lomustine + ^52^ TMZ vs. ^52^ TMZ	Primary	III	129	Health-related quality of life: No significant differences; Neurocognitive function: Mini-mental state examination (favors the TMZ group); Neurocognitive test battery: No significant differences.	[139]
^54^Vistusertib + ^52^TMZ	Recurrent	I	15	Tolerability: Vistusertib 125 mg b.i.d. + TMZ 150 mg/m^2^ for 5 days; PFS-6: 26.6%; AEs: G1/G2.	[140]
^55^ Ascorbate + RT + ^52^ TMZ	Primary	I	11	DLTs: None; AEs: Dry mouth and chills; Median PFS: 9.4 months; Median OS: 18 months.	[141]
^56^ Plerixafor	Primary	I/II	9+20	Tolerability: No drug-attributable G3 toxicities; Median OS: 21.3 months (95% CI, 15.9-NA); PFS: 14.5 months (95% CI, 11.9-NA).	[142]
^16^ Depatux-M (+^52^ TMZ) vs. ^52^ TMZ/ ^2^ lomustine	Recurrent	II	260	Efficacy: Monotherapy is comparable to control (hazard ratio: 1.04, 95% CI, 0.73–1.48); Toxicities: Reversible corneal epitheliopathy; AEs: G3–G4 (25–30%)	[143]
^57^ VB-111 + ^12^ bevacizumab vs. ^12^ bevacizumab	Recurrent	III	256	Median OS: 6.8 months (combination) vs. 7.9 months (control); ORR: 27.3% (combination) vs. 21.9% (control); AEs (G3–G5): 67% (combination) vs. 40% (control).	[144]

^1^ Aurora kinase inhibitor; ^2^ Nitrosourea, also known as CCNU; ^3^ Proteasome inhibitor; ^4^ Taxane-derived antineoplastic agent; ^5^ Pan-class I phosphoinositide 3-kinase inhibitor; ^6^ Receptor tyrosine kinase inhibitor; ^7^ Topoisomerase I inhibitor; ^8^ Histone deacetylase inhibitor; ^9^ NMDA receptor inhibitor; ^10^ Phospholipid-interacting antimalarial drug; ^11^ Anti-diabetic drug ^12^ Anti-angiogenic agent; ^13^ Allogeneic/Autologous vaccine; ^14^ CDK4/6 inhibitor; ^15^ Poly ADP ribose polymerase (PARP) inhibitor; ^16^ Antibody–drug conjugate; ^17^ Third-generation nitrosourea; ^18^ Wee1 inhibitor; ^19^ Proteasome inhibitor; ^20^ Non-voltage-dependent calcium channel inhibitor; ^21^ Interferon-binding protein; ^22^ Rapamycin (mTOR) inhibitor; ^23^ Raf kinase and vascular endothelial growth factor receptor 2 inhibitor; ^24^ Angiopoietin blocking peptibody; ^25^ Histone deacetylase (HDAC) inhibitor; ^26^ Rapamycin (mTOR) inhibitor; ^27^ IGF-1R pathway modulator; ^28^ G protein-coupled receptor DRD2 antagonist; ^29, 30^ Monoclonal antibody; ^31^ Tyrosine kinase inhibitor; ^32^Farnesyltransferase inhibitor; ^33^ Tyrosine kinase inhibitor; ^34^ Vaccine; ^35^ Epidermal growth factor receptor (EGFR) inhibitor; ^36^ Pan-human EGRF tyrosine kinase inhibitor; ^37^ Human epidermal growth factor receptor 2 (HER2)-specific chimeric antigen receptor (CAR)-modified virus-specific T cells (VSTs); ^38^ Topoisomerase I inhibitor; ^39^ Oligodeoxynucleotide-containing unmethylated cytosine-guanosine motifs (C_p_G-ODN); ^40^ Recombinant human fusion protein; ^41^ Monovalent mesenchymal epithelial transition factor (MET) inhibitor; ^42^ Pan-VEGF receptor tyrosine kinase inhibitor; ^43^ Anti platelet-derived growth factor-α antibody; ^44^ Proteasome inhibitor; ^45^ Nitrosourea; ^46^ Platelet-derived growth factor receptor-β tyrosine kinase inhibitor; ^47^ Tyrosine kinase inhibitor; ^48^ Oral PI3K inhibitor; ^49^ AKT inhibitor; ^50^ Inhibitor of fibroblast growth factor receptor (FGFR) and vascular endothelial growth factor receptor (VEGFR); ^51^ Humanized anti-epidermal growth factor receptor (EGFR) antibody; ^52^ Alkylating agent; ^53^ Tyrosine kinase inhibitor; ^54^Dual mTORC1/2 inhibitor; ^55^ Causes oxidative stress; ^56^ Reversible C-X-C chemokine receptor type 4 (CXCR4) inhibitor; ^57^ Non-replicating adenovirus, also known as Ofranergene obadenovec. Abbreviations: RT: radiotherapy; CI: confidence Interval; OS: overall survival; OS-6: overall survival at 6 months; OS-12: overall survival at 12 months; AEs: adverse events; median PFS: median progression-free survival; PFS-3: progression-free survival at 3 months; PFS-6: progression-free survival at 6 months; ALT: alanine aminotransferase; ORR: overall response rate; DLTs: dose-limiting toxicity; MTD: maximum tolerated dose; b.i.d.: twice a day; MRD: minimal residual disease; TMZ: temozolomide; G1: grade 1; G2: grade 2; G3: grade 3; G4: grade 4; TRTs: treatment-related toxicities; TTP: time to progression; BBB: blood–brain barrier.

**Table 2:** Dosage regimen of approved drugs for the treatment of adult glioblastoma. (Source: Dailymed/ Food and Drug Administration (FDA) label). RT: radiotherapy.

Therapeutic Agent	Disease Type	Dosage Regimen
Temozolomide (TMZ)	Newly diagnosed	Concurrent: 75 mg/m^2^ daily for six weeks with focal RT. Adjuvant *: Starts followed by a 4-week rest period after concurrent therapy. 1st cycle, 150 mg/m^2^ daily for five days in a 28-day cycle. 150–200 mg/m^2^ daily for 5 days in a 28-day cycle, 2nd–6th cycles.
Bevacizumab	Recurrent	10 mg/kg as intravenous infusion every 2 weeks **.
Carmustine (BiCNU) for injection	-	150–200 mg/m^2^ (single or divided into two successive days) intravenously every 6 weeks.
Carmustine (BiCNU) implant	Newly diagnosed/Recurrent	Eight 7.7 mg wafers with a total of 61.6 mg implanted intracranially.

* Dose could be reduced based on the appearance of toxicity. ** Treatment to be continued until disease progression or unacceptable toxicity.

## Supplementary Materials

The following are available online at https://www.mdpi.com/2072-6694/12/4/937/s1, Table S1: Ongoing clinical trials of targeted therapeutic agents summarized from www.clinicaltrials.gov. The details are collected from corresponding clinical trials and www.cancer.gov. The superscript numbers are unique for the therapeutic agents listed in Table S1 and are used to identify an agent in case of repeated presence in the table. They are unrelated to the superscript numbering used in Table 1.
