# Supplementary material for: Glioblastoma: Pathogenesis and Current Status of Chemotherapy and Other Novel Treatments
Source: Cancers (Basel). 2020 Apr 10;12(4):937. doi: 10.3390/cancers12040937 (PMC7226351; doi:10.3390/cancers12040937)
Supplement: Supplementary file 1 [file cancers-12-00937-s001.pdf]

Supplementary Materials

Glioblastoma: Pathogenesis and Current Status of Chemo and Other Novel Therapies

Vilashini Rajaratnam, Mohammad Mohiminul Islam, Maixee Yang, Rachel Slaby, Hilda Martinez Ramirez, and Shama Parveen Mirza

Table S1. Ongoing clinical trials of targeted therapeutic agents summarized from www.clinicaltrials.gov. The detains are collected from corresponding clinical trials and www.cancer.gov. The superscript numbers are unique for the therapeutic agents listed in Table S1 and are used to identify an agent in case of repeated presence in the table. They are unrelated to the superscript numbering used in Table 1.

Therapeutic Agent (s) under Investigation	Description/Mechanism of Action	Disease Type	Clinical Trial Phase	Estimated Completion Date	Clinical Trial Identifier
[1]2-OHOA	[1]2-hydroxy oleic acid, activates sphingomyelin synthase	Primary	I	September 2023	NCT03867123
[2]5-Flucytosine, [3]TG6002	[2]Fluorinated cytosine analog, inhibits thymidylate synthetase; [3]Oncolytic virus	Recurrent	I/II	September 2021	NCT03294486
[4]9-ING-41	[4]GSK-3β inhibitor	-	I/II	November 2022	NCT03678883
[5]ABBV-321	[5]Antibody-drug conjugate, targets EGFR	-	I	August 2023	NCT03234712
[6]Abemaciclib	[6]CDK inhibitor	Recurrent	II	April 2022	NCT02981940
[6]Abemaciclib, [7]CC-115, [8]Neratinib	[7]DNA-pk and mTOR inhibitor; [8]Tyrosine kinase inhibitor	-	II	May 2021	NCT02977780
[9]ABI-009	[9]mTOR inhibitor	Primary	II	June 2021	NCT03463265
[10]ACP-196	[10]Bruton's tyrosine kinase (Btk) inhibitor	Recurrent	Ib/II	December 2019	NCT02586857
[11]Adavosertib	[11]Trosine kinase WEE1 inhibitor	Primary	I	October 2021	NCT01922076
[11]Adavosertib	-	Recurrent	I	February 2020	NCT01849146
[12]Ad-hCMV-TK, [13]AdhCMV-Fit3L	[12]Adenoviral vector engineered to express HSV-tk; [13]Adenoviral vector engineered to express Flt3L	Primary	I	December 2020	NCT01811992
[14]Ad-RTS-hIL-12	[14]Adenoviral vector encoding hIL-12	Recurrent	I	December 2019	NCT02026271

Therapeutic Agent (s) under Investigation	Description/Mechanism of Action	Disease Type	Clinical Trial Phase	Estimated Completion Date	Clinical Trial Identifier
[14]Ad-RTS-hIL-12	-	-	I	March 2021	NCT03330197
[14]Ad-RTS-hIL-12, [15]Nivolumab, [16]Veledimex	[15]Immune checkpoint inhibitor that binds to PD-1; [16]Diacylhydrazine-based activator ligand that binds to RheoSwitch Therapeutic System (RTS)	Recurrent	I	December 2021	NCT03636477
[17]ADV/HSV-tk	[17]Gene therapy	Primary	I/II	December 2025	NCT03603405
[17]ADV/HSV-tk	-	Recurrent	I	December 2025	NCT03596086
[18]AdV-tk, [15]Nivolumab, [19]Temozolomide, [20]Valacyclovir	[18]Gene therapy; [19]DNA Alkylating agent; [20]Antiviral prodrug, inhibits viral DNA polymerase	Primary	I	January 2022	NCT03576612
[21]Afatinib	[21]Tyrosine kinase inhibitor	Recurrent	I	December 2020	NCT02423525
[22]Aldesleukin, [23]Cyclophosphamide, [24]Fludarabine	[22]Recombinant human interleukin-2; [23]Synthetic DNA alkylating agent; [24]DNA polymerase inhibitor	-	II	March 2028	NCT03412877
[22]Aldesleukin, [23]Cyclophosphamide, [24]Fludarabine, [25]Pembrolizumab, [26]Young TIL	[25]Humanized monoclonal antibody, inhibitor of PD-1 receptor; [26]TIL: Therapeutic tumor infiltrating lymphocyte	-	II	December 2024	NCT01174121
[27]ALECSAT	[27]ALECSAT: Autologous lymphoid effector cells specific against tumor	Primary	II	June 2021	NCT02799238
[28]Alectinib, [29]APG101, [30]Atezolizumab, [31]Idasanutlin, [32]Palbociclib, [33]Temsirolimus, [34]Vismodegib	[28]Tyrosine kinase inhibitor; [29]inhibitor of CD95L from binding to CD95 receptor; [30]Humanized monoclonal antibody; [31]Antagonist of MDM2 (mouse double minute 2); [32]Cyclin-dependent kinase inhibitor; [33]mTOR inhibitor; [34]Antagonist of the Hedgehog-ligand cell surface receptors PTCH and/or SMO	Primary	I/II	September 2024	NCT03158389

Therapeutic Agent (s) under Investigation	Description/Mechanism of Action	Disease Type	Clinical Trial Phase	Estimated Completion Date	Clinical Trial Identifier
[35](Allogeneic Hematopoietic Stem Cells, Cytotoxic Lymphocytes, Dendritic Vaccine)	[35]Proteome based personalized immunotherapy	-	II/III	December 2020	NCT01759810
[36]AMG 596	[36]Anti-EGFRvIII/CD3 BiTE antibody	Primary/ Recurrent	I	April 2021	NCT03296696
[37]AMG-232	[37]MDM2 inhibitor	Primary/ Recurrent	I	June 2020	NCT03107780
[38]Anti-EGFRvIII CAR T cells, [23]Cyclophosphamide, [24]Fludarabine	[38]Anti-EGFRvIII CAR-transduced allogeneic T lymphocytes	Recurrent	I	July 2019	NCT02844062
[39]Antigen-specific IgT cells	[39]Immunogene-modified T cells	-	I	December 2020	NCT03170141
[40]Anti-PD-L1 CSR T Cells, [23]Cyclophosphamide, [24]Fludarabine	[40]Autologous chimeric switch receptor modified T cells	Recurrent	I	July 2019	NCT02937844
[41]Apatinib	[41]Tyrosine kinase inhibitor	-	-	October 2019	NCT03567135
[42]Aprepitant, [43]Minocycline, [44]Disulfiram, [45]Celecoxib, [46]Sertraline, [47]Captopril, [48]Itraconazole, [49]Ritonavir, [50]Auranofin	[42]Substance P inhibitor; [43]Inhibits binding tRNA to the ribosomal complex, inhibits the enzyme 5-lipoxygenase (5LOX); [44]Proteasome inhibitor; [45]Cyclo-oxygenase (COX-2) inhibitor; [46]Selective serotonin reuptake inhibitor; [47]Angiotensin converting enzyme inhibitor; [48]Inhibitor of fugal cytochrome P450 enzymes; [49]Anti-HIV and a type of protease inhibitor; [50]Inhibits the activity of mitochondrial thioredoxin reductase (TrxR)	Recurrent	I	March 2020	NCT02770378
[51]APX005M	[51]CD40 agonistic monoclonal antibody	Recurrent	I	September 2022	NCT03389802

Therapeutic Agent (s) under Investigation	Description/Mechanism of Action	Disease Type	Clinical Trial Phase	Estimated Completion Date	Clinical Trial Identifier

[52]Arsenic trioxide	[52]Damages the promyelocytic leukemia protein/ retinoic acid receptor-alpha fusion protein	-	I/II	May 2021	NCT00275067
[53]Ascorbate	[53]Causes oxidative stress	-	I	December 2021	NCT01752491
[53]Ascorbic Acid	-	-	II	December 2023	NCT02344355
[54]Atezolizumab	[54]Humanized monoclonal antibody, inhibits PD-L1 from binding to PD-1	Primary	I/II	June 2020	NCT03174197
[54]Atezolizumab, [55]Ipatasertib	[55]Inhibitor of protein kinase B	-	I	November 2020	NCT03673787
[54]Atezolizumab, [56]PVSRIPO	[56]Polio/Rhinovirus Recombinant	Recurrent	I/II	July 2023	NCT03973879
[57]Avelumab	[57]Human IgG1 monoclonal antibody, inhibits interaction of PD-L1 with PD-1	-	II	February 2020	NCT02968940
[57]Avelumab	-	Primary	II	September 2022	NCT03047473
[57]Avelumab	-	Recurrent	I	February 2020	NCT03341806
[57]Avelumab, [58]Epacadostat	[58]Inhibitor of indoleamine 2,3-dioxygenase (IDO1)	Recurrent	II	April 2024	NCT03532295
[57]Avelumab, [59]VXM01	[59]Vascular endothelial growth factor receptor 2 (VEGRF-2) vaccine	Recurrent	I/II	December 2020	NCT03750071
[60]AV-GBM-1	[60]Autologous tumor-associated antigens (TAAs)-loaded autologous dendritic cells	Primary	II	June 2023	NCT03400917
[61]AZD1390	[61]ATM inhibitor	-	I	April 2021	NCT03423628
[62]AZD2014	[62]mTOR inhibitor	Recurrent	I	December 2019	NCT02619864
[63]BAL101553	[63]Prevents tubulin polymerization and destabilizes microtubules	Primary	I	July 2022	NCT03250299
[63]BAL101553	-	Primary	I	July 2022	NCT03250299
[63]BAL101553	-	Recurrent	I/IIa	December 2019	NCT02895360, NCT02490800
[64]Bavituximab	[64]IgG3 monoclonal antibody, stimulates antibody-dependent cellular cytotoxicity	Primary	II	July 2023	NCT03139916
**Therapeutic Agent (s) under Investigation**	**Description/Mechanism of Action**	**Disease Type**	**Clinical Trial Phase**	**Estimated Completion Date**	**Clinical Trial Identifier**
[65]Belinostat	[65]Hydroxamic acid-type histone deacetylase inhibitor	Primary	II	June 2022	NCT02137759

Therapeutic Agent (s) under Investigation	Description/Mechanism of Action	Disease Type	Clinical Trial Phase	Estimated Completion Date	Clinical Trial Identifier
[66]Bevacizumab	[66]Recombinant humanized monoclonal antibody, inhibits VEGF receptor binding	Primary	II	January 2021	NCT01149850
[66]Bevacizumab	-	Recurrent	II	September 2023	NCT01730950
[66]Bevacizumab	-	Primary	II	December 2019	NCT01740258
[66]Bevacizumab	-	Recurrent	II	December 2020	NCT01478321
[66]Bevacizumab	-	Recurrent	I	July 2020	NCT01392209
[66]Bevacizumab	-	Recurrent	II	December 2019	NCT00337207
[66]Bevacizumab	-	-	I	June 2020	NCT02285959
[66]Bevacizumab	-	Recurrent	II	March 20202	NCT02663271
[66]Bevacizumab	-	Recurrent	II	December 2019	NCT01894061
[66]Bevacizumab	-	Primary	I/II	January 2020	NCT01811498
[66]Bevacizumab	-	Recurrent	I/II	October 2021	NCT01269853
[66]Bevacizumab, [67]Capecitabine	[67]Fluoropyrimidine carbamate, prodrug of 5-fluorouracil	Recurrent	I	November 2019	NCT02669173
[66]Bevacizumab, [68]Keyhole Limpet Hemocyanin, Peptide Vaccine, [69]PolyICLC	[68]Immunogenic carrier protein, increases antigenic immune responses; [69]Synthetic complex of carboxymethylcellulose, polyinosinic-polycytidylic acid, and poly-L-lysine double-stranded RNA	Recurrent	II	June 2019	NCT02754362
[66]Bevacizumab, [15]Nivolumab	-	Recurrent	II	December 2019	NCT03452579
[66]Bevacizumab, [19]Temozolomide	-	Recurrent	III	July 2023	NCT02761070
[70]BGB-290	[70]Inhibitor of poly (ADP-ribose) polymerase (PARP)	Primary/ Recurrent	I	July 2027	NCT03749187
[70]BGB-290	-	Primary/ Recurrent	I/II	October 2021	NCT03150862
[70]BGB-290	-	Primary/ Recurrent	I	July 2027	NCT03749187

[71]BGB324	[71]AXL kinase inhibitor	Recurrent	I	December 2022	NCT03965494
[72]BKM120	[72]Pan class-1 phosphoinositide 3-kinase inhibitor	Recurrent	I/II	February 2020	NCT01349660
[73]BMS 986016	[73]Monoclonal antibody, binds to lymphocyte activation gene-3 (LAG-3) receptor	Recurrent	I	December 2020	NCT02658981
[73]BMS-986016, [15]Nivolumab	-	Recurrent	I	June 2021	NCT03493932
[74]BMX-001	[74]Mimetic of the human mitochondrial manganese superoxide dismutase (MnSOD)	Primary	II	September 2022	NCT02655601
[75]Bortezomib	[75]26S proteasome inhibitor	Recurrent	Ib/II	August 2023	NCT03643549
[76]BPM31510	[76]Modulates tumor cell metabolism	Recurrent	I	January 2021	NCT03020602
[77]BRCX014	[77]Cannabidiol	Primary	I	December 2020	NCT03687034
[77]BRCX014	-	Primary	II	December 2020	NCT03607643
[78]BXQ-350	[78]Synthetic form of the human glycoprotein saposin C (SapC), activates lysosomal enzymes	Recurrent	I	January 2020	NCT02859857
[79]C134	[79]Oncolytic virus HSV-1	Recurrent	I	May 2024	NCT03657576
[80]C225-ILs-dox	[80]Doxorubicin-loaded Anti-EGFR-immunoliposomes	Recurrent	I	August 2021	NCT03603379
[81]Cabozantinib	[81]Tyrosine kinase inhibitor	Recurrent	II	January 2020	NCT02885324
[82]CAN008	[82]Antibody like CD95 receptor/Fc fusion protein	Recurrent	II	December 2021	NCT03746288
[67]Capecitabine	-	Primary	I/II	June 2021	NCT03213002
[83]Carboplatin	[83]Second generation platinum compound, induces intra-strand and inter-strand DNA cross-links	Recurrent	I/II	July 2021	NCT03672721
[84]Carboxyamidotriazole (CTO)	[84]Inhibitor of non-voltage operated Ca^{2+} channels	Primary	I	-	NCT01107522
[85]Carboxylesterase-expressing allogeneic neural stem cells, [86]Irinotecan hydrochloride	[85]Human fetal cell line adenovirally transduced to express a modified form of the enzyme carboxylesterase hCE1m6; [86]Prodrug, converts into active metabolite 7-ethyl-10-hydroxy-camptothecin which inhibits topoisomerase I	Recurrent	I	March 2020	NCT02192359

**Therapeutic Agent (s) under Investigation**	**Description/Mechanism of Action**	**Disease Type**	**Clinical Trial Phase**	**Estimated Completion Date**	**Clinical Trial Identifier**

[87]Carcinoembryonic Antigen-Expressing Measles Virus	[87]Oncolytic viral therapy	Recurrent	I	November 2019	NCT00390299
[88]CART-EGFRvIII T cells, [25]Pembrolizumab	[88]Engineered autologous T cells with specificity for EGFRvIII	Primary	I	December 2034	NCT03726515
[89]CBL0137	[89]Curaxin-based agent that targets Facilitates Chromatin Transcription (FACT) complex	-	I	November 2019	NCT01905228
[90]CC-115	[90]mTOR and DNA-PK inhibitor	-	I	November 2019	NCT01353625
[91]CC-122	[91]Cereblon modulator	-	I	August 2020	NCT01421524
[92]CC-486	[92]Inhibits DNA methyltransferase, and thus blocks DNA methylation	-	I	December 2018	NCT02223052
[93]Cediranib	[93]Inhibitor of vascular endothelial growth factor receptor (VEGFR-1,-2,-3) tyrosine kinases	Primary	II	-	NCT01062425
[93]Cediranib Maleate, [94]Olaparib	[94]Inhibitor of poly (ADP-ribose) polymerase	Recurrent	II	May 2020	NCT02974621
[95]Cemiplimab, [96]INO-5401, [97]INO-9012	[95]Inhibits PD-1 from binding to PD-L1; [96]WT1/PSMA/hTERT encoding plasmid DNA; [97]DNA plasmid encoding interleukin-12	Primary	I/II	January 2021	NCT03491683
[98]Cerebraca Wafer	[98]Biodegradable implant comprises (Z)-n-butylidenephthalide ((Z)-BP) and carboxyphenoxypropane-sebacic acid	Recurrent	I/IIa	December 2021	NCT03234595
[99]Cetuximab, [100]Mannitol	[99]Anti-EGFR monoclonal antibody; [100]Osmotic diuretic	Primary	I/II	June 2021	NCT02861898
[99]Cetuximab, [100]Mannitol	-	Recurrent	II	May 2022	NCT02800486
[101]Chloroquine	[101]Chemosensitizer, inhibits autophagy	Primary	II	January 2024	NCT02432417
[101]Chloroquine	-	Primary	I	June 2019	NCT02378532

**Therapeutic Agent (s) under Investigation**	**Description/Mechanism of Action**	**Disease Type**	**Clinical Trial Phase**	**Estimated Completion Date**	**Clinical Trial Identifier**
[101]Chloroquine, [19]Temozolomide, [102]Valproic Acid	[102]Anti-epileptic drug, antitumor activity is assumed to related to inhibition of nitric oxide synthesis	-	III	June 2023	NCT03243461

[103]CMV-Specific T Cells	[103]Autologous cells, CMV = Cytomegalovirus	-	I/II	June 2020	NCT02661282
Copper, [44]Disulfiram	-	Primary	II	March 2021	NCT03363659
Copper, [44]Disulfiram	-	Primary	I/II	April 2021	NCT02715609
Copper, [44]Disulfiram	-	Recurrent	II/II	September 2023	NCT02678975
[104]Crenolanib	[104]Inhibitor of platelet-derived growth factor receptor (PDGFR)	Recurrent	II	April 2020	NCT02626364
[23]Cyclophosphamide, [105]ERC1671, [106]GM-CSF	[105]Cancer vaccine, induces cytotoxic T-lymphocity (CTL) response against GBM-associated antigens; [106]Recombinant agent similar to endogenous human GM-CSF, modulates proliferation and differentiation	Recurrent	II	March 2023	NCT01903330
[23]Cyclophosphamide, [107]Etoposide, [108]Indoximod	[107]Topoisomerase II inhibitor; [108]Immune checkpoint inhibitor	-	I	December 2019	NCT02502708
[23]Cyclophosphamide, [109]rQNestin	[109]Oncolytic HSV-1	Recurrent	I	July 2022	NCT03152318
[110]Dabrafenib, [111]Trametinib	[110]B-raf (BRAF) protein inhibitor; [111]Inhibitor of mitogen-activated protein kinase (MEK MAPK/ERK kinase)	Primary	II	June 2022	NCT03919071
[110]Dabrafenib, [111]Trametinib	-	Recurrent	II	September 2024	NCT02684058
[112]Dasatinib	[112]Inhibitor of SRC-family protein-tyrosine kinases	Primary	I/II	-	NCT00869401
[113]Dendritic Cell Vaccination	[113]Immunotherapy (therapeutic autologous dendritic cells)	Primary	II	December 2023	NCT03927222
[113]Dendritic Cell Vaccination	-	-	I	November 2023	NCT01808820
[113]Dendritic Cell Vaccination	-	Primary/ Recurrent	I	January 2021	NCT02010606
**Therapeutic Agent (s) under Investigation**	**Description/Mechanism of Action**	**Disease Type**	**Clinical Trial Phase**	**Estimated Completion Date**	**Clinical Trial Identifier**
[113]Dendritic Cell Vaccination	-	Recurrent	I/II	January 2022	NCT03879512
[113]Dendritic Cell Vaccination	-	Primary	II	June 2020	NCT02366728

[113]Dendritic Cell Vaccine	-	Primary	I/II	December 2022	NCT02649582
[113]Dendritic Cells	-	Primary	II	June 2024	NCT02465268
[113]Dendritic Cells, [114]Lymphocytes, [115]Tetanus Toxoid	[114]Immunotherpay (therapeutic autologous lymphocytes); [115]Stimulates the production of antitoxin antibodies	Primary	I	January 2020	NCT00639639
[113]Dendritic Cells, [15]Nivolumab	-	Recurrent	II	June 2022	NCT03014804
[113]Dendritic Cells, [115]Tetanus toxoid	-	Primary	I	August 2020	NCT03615404
[113]Dendritic Cells	-	-	II/II	May 2023	NCT03548571
[113]Dendritic Cells	-	Primary	II	June 2023	NCT03395587
[44]Disulfiram, Copper	-	Primary	II	January 2020	NCT01777919
[116]DNX-2401	[116]Oncolytic adenovirus serotype 5 strain	Recurrent	I	February 2020	NCT03896568
[116]DNX-2401, [25]Pembrolizumab	-	Recurrent	II	June 2021	NCT02798406
[117]DNX-2440	[117]OX40L expressing oncolytic adenovirus	Recurrent	I	October 2022	NCT03714334
[118]Doxorubicin	[118]Intercalates between base pairs, inhibits topoisomerase II, and thus prevents DNA replication	-	II	July 2020	NCT02758366
[118]Doxorubicin, [107]Etoposide	-	-	I	October 2020	NCT02372409
[119]DSP-7888	[119]Wilms tumor gene 1 (WT1) protein derived peptide vaccine	Recurrent	I/II	April 2020	NCT02750891
[119]DSP-7888	-	Recurrent	II	October 2020	NCT03149003

Therapeutic Agent (s) under Investigation	Description/Mechanism of Action	Disease Type	Clinical Trial Phase	Estimated Completion Date	Clinical Trial Identifier
[120]Durvalumab	[120]Monoclonal antibody, blocks PD-L1 from binding to PD-1	Recurrent	I/II	July 2020	NCT02866747
[121]EGFR BATs	[121]EGFRBi-armed activated T cells, induces T cell mediated cytotoxicity	Primary	I	December 2020	NCT03344250
[122]EGFR(V)-EDV-Dox	[122]Doxorubicin-loaded EGFR-targeting nanocells, inhibits topoisomerase II, and thus inhibits DNA replication	Recurrent	I	June 2020	NCT02766699

Therapeutic Agent (s) under Investigation	Description/Mechanism of Action	Disease Type	Clinical Trial Phase	Estimated Completion Date	Clinical Trial Identifier
[88]EGFRvIII CAR T Cells	-	Primary	I	September 2019	NCT02664363
[88]EGFRvIII-CAR-T Cells	-	Recurrent	I	December 2022	NCT03283631
[123]Enzastaurin	[123]Inhibitor of protein kinase C beta	Primary	IIb	December 2022	NCT03776071
[124]Epitinib Succinate (HMPL-813)	[124]Inhibitor of epidermal growth factor receptor	-	I	August 2020	NCT03231501
[125]Erbitux, [66]Bevacizumab	[125]Inhibitor of epidermal growth factor receptor	Recurrent	I/II	January 2025	NCT01884740
[126]Everolimus (RAD001)	[126]mTOR inhibitor	Primary	I/II	-	NCT01062399
[126]Everolimus (RAD001)	-	Primary	I/II	-	NCT00553150
[126]Everolimus, [127]Ribociclib	[127]Inhibitor of cyclin-dependent kinase (CDK)	Recurrent	I	December 2020	NCT03834740
[126]Everolimus, [127]Ribociclib	-	Primary	I	January 2022	NCT03355794
[126]Everolimus, [128]Sorafenib	[128]Inhibitor of RAF kinase and VEGFR-2/PDGFR-beta signaling cascade	Recurrent	I/II	July 2020	NCT01434602
[129]Filgrastim, [130]Gemcitabine, [127]Ribociclib, [131]Sonidegib, [132]Trametinib	[129]Agonist of G-CSF receptor; [130]Inhibitor of ribonucleotide reductase; [131]Inhibitor of hedgehog (Hh)-ligand cell surface receptor Smo; [132]Inhibitor of mitogen-activated protein kinase kinase	Recurrent	I	March 2025	NCT03434262
[133]Fimepinostat	[133]Inhibitor of phosphoinositide 3-kinase (PIK) class I and pan histone deacetylase (HDAC)	Recurrent	I	June 2027	NCT03893487
[2]Flucytosine, [134]Leucovorin Calcium, [135]Neural Stem Cells	[134]Active metabolite of folic acid, counteracts the toxic effect of folate antagonist-type chemotherapeutic drugs; [135]Genetically modified E. coli cytosine deaminase (CD)-expressing neural stem cells	Recurrent	I	June 2019	NCT02015819
[136]FT-2102	[136]IDH-1 inhibitor	-	I	April 2022	NCT03684811
[137]GC1118	[137]Anti-EGFR monoclonal antibody	Recurrent	II	July 2020	NCT03618667
[138]GDC-0084	[138]Phosphatidylinositol 3-kinase (PI3K) inhibitor	Primary	II	December 2020	NCT03522298
[138]GDC-0084	-	Primary	I	June 2024	NCT03696355

[139]Glasdegib (PF-04449913)	[139]Smoothened (SMO) receptor inhibitor	Primary	Ib/II	March 2021	NCT03466450
[140]gp96	[140]Autologous heat shock protein	Primary	II	August 2023	NCT03650257
[141]GS5745	[141]Anti-MMP-9 monoclonal antibody	Recurrent	I	January 2022	NCT03631836
[141]GS5745	-	Recurrent	I	January 2022	NCT03631836
[142]GX-I7	[142]Recombinant human IL-7-hybrid Fc	-	I/II	May 2020	NCT03619239
[143]hrBMP4	[143]Recombinant human bone morphogenetic protein 4, binds to BMP receptors (BMPRs)	Recurrent	I	August 2019	NCT02869243
[144]HSPPC-96	[144]Autologous cancer vaccine derived from tumor-specific heat shock protein gp96	Primary/ Recurrent	I	May 2020	NCT02722512
[144]HSPPC-96, [25]Pembrolizumab	-	Primary	II	January 2024	NCT03018288
[145]G207	[145]Recombinant HSV-1, inhibits UL39 and viral ribonucleotide reductase	Recurrent	I	July 2023	NCT03911388
[145]G207	-	Recurrent	I	April 2020	NCT02457845
[146]Hydroxyurea	[146]Selective inhibitor of ribonucleoside diphosphate reductase	Recurrent	I	June 2020	NCT03463733
[147]Ibrutinib	[147]BTK inhibitor	Primary	I	December 2019	NCT03535350
[148]IDH305	[148]IDH1 (R132) inhibitor	-	I	June 2020	NCT02381886

**Therapeutic Agent (s) under Investigation**	**Description/Mechanism of Action**	**Disease Type**	**Clinical Trial Phase**	**Estimated Completion Date**	**Clinical Trial Identifier**
[149]IMMU-132	[149]Antibody-drug conjugate containing humanized monoclonal antibody, hRS7, against tumor-associated calcium signal transducer 2 and linked to the active metabolite of Irinotecan	-	I/II	June 2020	NCT01631552
[150]INC280	[150]Inhibitor of the proto-oncogene c-Met (hepatocyte growth factor receptor)	Recurrent	Ib	November 2020	NCT02386826
[151]INCB024360, [152]Ipilimumab,	[151]Inhibitor of indoleamine 2,3-dioxygenase (IDO1); [152]Recombinant human monoclonal antibody, inhibitor of	Recurrent	I	February 2024	NCT03707457

[153]MK-4166, [15]Nivolumab	CTLA4-mediated downregulation of T-cell activation; [153]Anti-human glucocorticoid-induced tumor necrosis factor receptor (GITR) agonistic monoclonal antibody				
[154]INT230-6	[154]Formulation of cisplatin, vinblastine and a cell penetration enhancer	Recurrent	I	August 2020	NCT03058289
[155]Lonafarnib	[155]Inhibitor of farnesyl transferase	Recurrent	I	December 2020	NCT00102648
[152]Ipilimumab, [15]Nivolumab	-	Primary	I	-	NCT02311920
[152]Ipilimumab, [15]Nivolumab	-	Recurrent	II	August 2021	NCT03430791
[152]Ipilimumab, [15]Nivolumab	-	Recurrent	I	November 2019	NCT03233152
[152]Ipilimumab, [15]Nivolumab	-	Primary	II	January 2020	NCT03367715
[86]Irinotecan	-	Recurrent	I/II	July 2020	NCT03119064
[156]KB004	[156]Anti ephrin receptor A3 (EphA3) monoclonal antibody	Recurrent	I	April 2021	NCT03374943
[157]Lapatinib	[157]Orally-active quinazoline, reversibly blocks phosphorylation of the epidermal growth factor receptor (EGFR), ErbB2, and the Erk-1 and-2 and AKT kinases	Primary	II	December 2022	NCT01591577

Therapeutic Agent (s) under Investigation	Description/Mechanism of Action	Disease Type	Clinical Trial Phase	Estimated Completion Date	Clinical Trial Identifier
[158]Lapatinib Ditosylate	[158]Ditosylate salt of lapatinib	Recurrent	I	December 2019	NCT02101905
[159]LB-100	[159]Protein phosphatase 2A inhibitor	Recurrent	II	August 2021	NCT03027388
[160]Lenvatinib, [25]Pembrolizumab	[160]Inhibitor of vascular endothelial growth factor receptor 2 tyrosine kinase	-	II	April 2022	NCT03797326
[161]Levetiracetam	[161]Anti-epileptic drug, inhibits burst firing without affecting normal neuronal excitability	Primary	II	June 2022	NCT02815410
[162]Lomustine	[162]Nitrosourea compound, alkylates and crosslinks DNA	Recurrent	II	November 2021	NCT03022578
[162]Lomustine, [163]Sunitinib	[163]Inhibits tyrosine kinase activities of vascular endothelial growth factor receptor 2, platelet-derived growth factor receptor b, and c-kit	Recurrent	II/III	January 2020	NCT03025893
[162]Lomustine, [164]USL311	[164]Inhibitor of C-X-C chemokine receptor type 4 (CXCR4)	Recurrent	I/II	September 2022	NCT02765165
[165]LOXO-195	[165]Tyrosine receptor kinase (TRK) inhibitor	-	I/II	February 2022	NCT03215511

Therapeutic Agent (s) under Investigation	Description/Mechanism of Action	Disease Type	Clinical Trial Phase	Estimated Completion Date	Clinical Trial Identifier
[166]LY2157299	[166]Inhibitor of the tyrosine kinase transforming growth factor-beta (TGF-b) receptor type 1	Recurrent	II	December 2019	NCT01582269
[167]M032	[167]Genetically engineered HSV-1 expressing IL-12	Recurrent	I	September 2020	NCT02062827
[168]Marizomib	[168]Inhibitor of the 20S catalytic core subunit of the proteasome	Primary	I	May 2020	NCT02903069
[168]Marizomib	-	Primary	III	July 2023	NCT03345095
[168]Marizomib	-	-	I/II	February 2020	NCT02330562
[169]MBG453	[169]Immunotherapy	Recurrent	I	June 2023	NCT03961971
[170]MDNA55	[170]IL4-Pseudomonas exotoxin fusion protein, binds to IL-4 receptors on tumor cell surface	Recurrent	II	March 2020	NCT02858895
[171]Mebendazole	[171]Anthelmintic, inhibits the formation cytoplasmic microtubules in helminths	Primary	I	September 2020	NCT01729260
[171]Mebendazole	-	-	I	April 2020	NCT01837862
[171]Mebendazole	-	Recurrent	I	June 2021	NCT02644291
[172]MEDI4736	[172]Fc optimized monoclonal antibody, prevents PD-L1 from binding to PD-1	Primary/ Recurrent	II	December 2019	NCT02336165
[173]Memantine, [174]Mefloquine, [175]Metformin	[173]N-methyl-D-asparatate receptor antagonist; [174]Chemosensitizer, accumulates in lysosome and disrupts lysosomal function, and assumed to inhibit autophagocytosis; [175]Antihyperglycemic, inhibits complex I (NADPH: ubiquinon oxidoreductase) of the mitochondrial respiratory chain	-	I	September 2022	NCT01430351
[175]Metformin	-	Recurrent	II	December 2019	NCT03243851
[175]Metformin	-	Primary	II	December 2021	NCT02780024
[175]Metformin	-	Recurrent	I	July 2018	NCT02149459
[176]MK-3475	[176]Humanized monoclonal antibody, inhibits PD-1 activation by its ligands	Recurrent	I	August 2021	NCT02852655
[177]MN-166	[177]Inhibitor of cyclic nucleotide phosphodiesterase	Recurrent	I/II	December 2020	NCT03782415

[178]NEO100	[178]Inhibitor of farnesyl transferase and geranylgeranyl transferase	Recurrent	I/II	October 2020	NCT02704858
[179]Neural Stem Cell Based Virotherapy	[179]Neural stem cells loaded with an oncolytic adenovirus	Primary	I	April 2020	NCT03072134
[15]Nivolumab	-	Primary	III	August 2023	NCT02667587
[15]Nivolumab	-	Recurrent	II	November 2021	NCT03743662
[15]Nivolumab	-	Recurrent	II	August 2022	NCT03890952
[15]Nivolumab	-	Primary	III	September 2020	NCT02617589
[15]Nivolumab	-	-	II	August 2025	NCT03718767
[15]Nivolumab, [58]Epacadostat	-	-	II	October 2020	NCT02327078
[15]Nivolumab, [152]Ipilimumab	-	Recurrent	III	October 2019	NCT02017717

**Therapeutic Agent (s) under Investigation**	**Description/Mechanism of Action**	**Disease Type**	**Clinical Trial Phase**	**Estimated Completion Date**	**Clinical Trial Identifier**
[180]NK-92/5.28.z Cells	[180]Genetically modified anti-HER2-CAR-CD28zeta-expressing allogeneic natural killer cells, binds to HER2 expressed on tumor cells	Recurrent	I	August 2020	NCT03383978
[181]NU-0129	[181]Spherical nucleic acid nanoparticle, inhibitor of Bcl-2-like protein 12 (BCL2L12) gene	Recurrent	I	July 2022	NCT03020017
[182]NVX-108	[182]Radiosensitizer, increases tumor oxygenation	Primary	II	December 2023	NCT03862430
[183]OKN-007	[183]Assumed to inhibit sulfatase-2 (SULF2)	Recurrent	I/II	December 2020	NCT03649464
[183]OKN-007	-	Primary	I	June 2025	NCT03587038
[93]Olaparib	-	-	II	July 2020	NCT03212274
[184]ONC201	[184]Inhibitor of the serine/threonine protein kinase Akt (protein kinase B) and extracellular signal-regulated kinase (ERK)	Recurrent	II	December 2020	NCT02525692
[185]PAC-1	[185]Procaspase activating compound-1	-	I	June 2020	NCT03332355
[186]Pazopanib	[186]Selective inhibitor of vascular endothelial growth factor receptors (VEGFR)-1,-2 and -3, c-kit and platelet derived growth factor receptor (PDGF-R)	Primary	I/II	March 2021	NCT02331498
[25]Pembrolizumab	-	Primary	I	November 2020	NCT02530502

[25]Pembrolizumab	-	Recurrent	II	November 2019	NCT02337686
[25]Pembrolizumab	-	Recurrent	II	February 2021	NCT03661723
[25]Pembrolizumab	-	Primary	II	February 2023	NCT03405792
[25]Pembrolizumab	-	Recurrent	I/II	February 2020	NCT03277638
[25]Pembrolizumab, [187]TTAC-0001	[187]Human monoclonal antibody, prevents vascular endothelial growth factor receptor 2 from binding to its ligand VEGF	Recurrent	Ib	April 2020	NCT03722342
[25]Pembrolizumab, [188]Vorinostat	[188]Inhibitor of histone deacetylase	Primary	I	April 2022	NCT03426891
[189]Pemetrexed Disodium	[189]Inhibitor of thymidylate synthase	Recurrent	II	December 2020	NCT00276783
[190]Perifosine, [33]Temsirolimus	[190]Alkyl-phosphocholine compound, involved in cell differentiation and inhibition of cell growth, also inhibits anti-apoptotic MAPK pathway	Recurrent	I	August 2019	NCT02238496

Therapeutic Agent (s) under Investigation	Description/Mechanism of Action	Disease Type	Clinical Trial Phase	Estimated Completion Date	Clinical Trial Identifier
[191]Personalized cellular Vaccine	[191]Personalized antigen pulsed DCs	Recurrent	I	September 2020	NCT02808364
[192]Plerixafor	[192]Inhibits the binding of stromal cell-derived factor (SDF-1alpha) to the cellular receptor CXCR4	Primary	II	January 2027	NCT03746080
[193]PLX3397	[193]Receptor tyrosine kinase (RTK) inhibitor of KIT, CSF1R and FLT3	Primary	Ib/II	October 2019	NCT01790503
[69]Poly-ICLC	-	Primary	I	May 2021	NCT03223103
[194]PT2385	[194]Inhibitor of hypoxia inducible factor (HIF)-2alpha	Recurrent	II	December 2019	NCT03216499
[195]PT2977	[195]Inhibitor of hypoxia inducible factor (HIF)-2alpha	-	I	June 2020	NCT02974738
[56]PVSRIPO	-	Recurrent	Ib	July 2021	NCT03043391
[56]PVSRIPO	-	Recurrent	II	May 2023	NCT02986178
[56]PVSRIPO	-	Recurrent	I	June 2021	NCT01491893
[196]Ramipril	[196]Inhibitor of nonsulfhydryl angiotensin converting enzyme (ACE)	-	II	August 2021	NCT03475186

[197]Regorafenib	[197]Inhibitor of vascular endothelial growth factor receptors (VEGFRs) 2 and , and Ret, Kit, PDGFR and Raf kinases	Recurrent	II	October 2018	NCT02926222
[198]Rhenium	[198]Liposomal rhenium 186, releases radiation, which kills tumor cells	Recurrent	I	January 2020	NCT01906385
[127]Ribociclib (LEE011)	-	Recurrent	I	January 2020	NCT02345824
[199]Rovalpituzumab Tesirine	[199]Antibody (SC16)-drug (D6.5) conjugate	-	I/II	September 2020	NCT02709889
[200]RRx-001	[200]Radiosensitizer	Primary	I	January 2020	NCT02871843
[201]Ruxolitinib	[201]Inhibitor of Janus-associated kinase (JAK)	Primary	I	December 2020	NCT03514069
[202]Sapanisertib	[202]Inhibitor of raptor-mTOR and rictor-mTOR	Recurrent	I	December 2019	NCT02142803
[203]Selinexor	[203]Inhibitor of CRM1 (chromosome region maintenance 1 protein)	Recurrent	II	December 2019	NCT01986348
[203]Selinexor	-	Recurrent	I	March 2021	NCT02323880

**Therapeutic Agent (s) under Investigation**	**Description/Mechanism of Action**	**Disease Type**	**Clinical Trial Phase**	**Estimated Completion Date**	**Clinical Trial Identifier**
[204]Sildenafil Citrate, [128]Sorafenib tosylate, [102]Valproic Acid	[204]Selective inhibitor of cyclic guanosine monophosphate (cGMP)-specific type 5 phosphodiesterase	Recurrent	II	June 2021	NCT01817751
[205]Siroquine (JP001)	[205]Siroquine: Rapamycin (mTOR inhibitor) + Hydroxychloroquine sulfate	Primary	II/III	October 2023	NCT03008148
[206]Solriamfetol	[206]Wakefulness promoting drug, improves sleep, memory, fatigue, mood, or quality of life	Primary	II	January 2022	NCT03868943
[207]Sym004	[207]Anti-EGFR monoclonal antibody mixture, inhibits EGFR dependent tumor cell proliferation and metastasis	Recurrent	II	May 2020	NCT02540161
[208]T Cells (genetically modified)	[208]IL13Rα2-specific, hinge optimized, 41BB-costimulatory CAR/truncated CD19-expressing autologous T lymphocytes	Recurrent	I	May 2020	NCT02208362
[209]Temferon	[209]Immunotherapy consists of autologous CD34+ enriched hematopoietic stem and progenitor cells	-	I/IIa	December 2022	NCT03866109
[19]Temozolomide	-	-	III	April 2021	NCT03633552

[19]Temozolomide (intensified)	-	Primary	III	May 2023	NCT03663725
[210]Tesevatinib	[210]Receptor tyrosine kinase (RTK) inhibitor	Recurrent	II	December 2019	NCT02844439
[211]TG02	[211]Inhibitor of cyclin dependent kinase (CDK) subtypes 1, 2, 7 and 9, Janus associated kinase 2 (JAK2), FMS related tyrosine kinase 3 (FLT3, FLK2, STK1)	Recurrent	I/II	August 2022	NCT02942264
[211]TG02	-	Primary/ Recurrent	I	August 2020	NCT03224104
[212]TH-302	[212]Induces intra- and inter-strand DNA crosslinks	-	II	July 2019	NCT02342379
[213]TIL (Tumor-infiltrating T Lymphocyte)	[213]Transgenic cells	-	I	June 2020	NCT03347097

Therapeutic Agent (s) under Investigation	Description/Mechanism of Action	Disease Type	Clinical Trial Phase	Estimated Completion Date	Clinical Trial Identifier
[214]Tinostamustine	[214]Alkylating histone-deacetylase inhibitor	Primary	I	October 2020	NCT03452930
[215]TN-TC11G (THC+CBD)	[215]THC = Δ9-tetrahydrocannabinol, CBD = Cannabidiol	Primary	Ib	June 2020	NCT03529448
[216]Toca 511, [217]Toca FC	[216]Replication competent retroviral vector (gene therapy); [217]An extended release oral tablet that contains flucytosine (prodrug), inhibits thymidylate synthetase	Recurrent	II/III	March 2023	NCT02414165
[218]Topotecan	[218]Selectively stabilizes topoisomerase I-DNA covalent complexes, inhibiting relegation of topoisomerase I-mediated single-strand DNA breaks	-	II	December 2019	NCT01004874
[218]Topotecan, [219]Pazopanib	[219]Selective inhibitor of vascular endothelial growth factor receptors (VEGFR)-1, -2 and -3, c-kit and platelet derived growth factor receptor (PDGF-R)	Recurrent	II	September 2020	NCT01931098
[220]Trans Sodium Crocetinate	[220]Antihypoxic and radiosensitizing agent	Primary	III	June 2022	NCT03393000
[221]Trebananib	[221]Angiopoietin (Ang) 1 and 2 neutralizing peptibody	Recurrent	II	-	NCT01609790
[222]Tremelimumab, [120]Durvalumab	[222]Human immunoglobulin (Ig) G2 monoclonal antibody, inhibits cytotoxic T-lymphocyte associated protein 4 (CTLA4) mediated downregulation of T-cell activation	Recurrent	II	June 2020	NCT02794883

[187]TTAC-0001	-	Recurrent	II	November 2020	NCT03856099
[223]VAL-083	[223]DNA alkylating and cross-linking agent	Recurrent	II	June 2021	NCT02717962
[223]VAL-083	-	Primary	II	June 2020	NCT03050736
[102]Valproic Acid, [66]Bevacizumab	-	Primary	II	May 2019	NCT00879437
[224]VBI-1901	[224]Cancer vaccine that exerts a cytotoxic T-lymphocyte (CTL) immune response against CMV gB and pp65 expressing tumor cells	Recurrent	I	December 2019	NCT03382977

**Therapeutic Agent (s) under Investigation**	**Description/Mechanism of Action**	**Disease Type**	**Clinical Trial Phase**	**Estimated Completion Date**	**Clinical Trial Identifier**
[225]V-Boost	[225]Oral tablet which contains hydrolyzed GBM antigens and alloantigens	Recurrent	II	June 2020	NCT03916757
[16]Veledimex, [14]Ad-RTS-hIL-12	-	Recurrent	I	June 2021	NCT03679754
[226]Veliparib	[226]Inhibitor of poly (ADP-ribose) polymerase (PARP) -1 and -2	Primary	II/III	June 2022	NCT02152982
[226]Veliparib	-	Primary	II	October 2024	NCT03581292
[188]Vorinostat	-	Primary	I/II	-	NCT00731731
[188]Vorinostat	-	Primary	I/II	November 2020	NCT01189266
[188]Vorinostat	-	-	I	-	NCT00268385
[188]Vorinostat, [227]Isotretinoin	[227]Agonist of nuclear retinoic acid receptors (RARs)	Recurrent	I/II	November 2019	NCT00555399
[228]WP1066	[228]Inhibitor of Janus-associated Kinase 2 (JAK2)	Recurrent	I	July 2021	NCT01904123
[229]ZSP1602	[229]SMO protein inhibitor	-	I	December 2020	NCT03734913
[230]α-IFN	[230]Immunotherapeutic agent	Primary	III	December 2019	NCT01765088
[231]β-elemene	[231]Induces apoptosis and cell cycle arrest	Primary	III	May 2023	NCT02629757

© 2019 by the authors. Submitted for possible open access publication under the terms and conditions of the Creative Commons Attribution (CC BY) license (http://creativecommons.org/licenses/by/4.0/).
